# Supplementary figures and images for: ATN Classification and Machine-Learned Plasma Biomarker Phenotypes Reveal Distinct Alzheimer’s Pathology in a Population-Based Cohort
Source: medRxiv. 2026 Feb 3:2026.01.02.26343331. Originally published 2026 Jan 6. Preprint. [Version 4] doi: 10.64898/2026.01.02.26343331 (PMC12803393; doi:10.64898/2026.01.02.26343331)

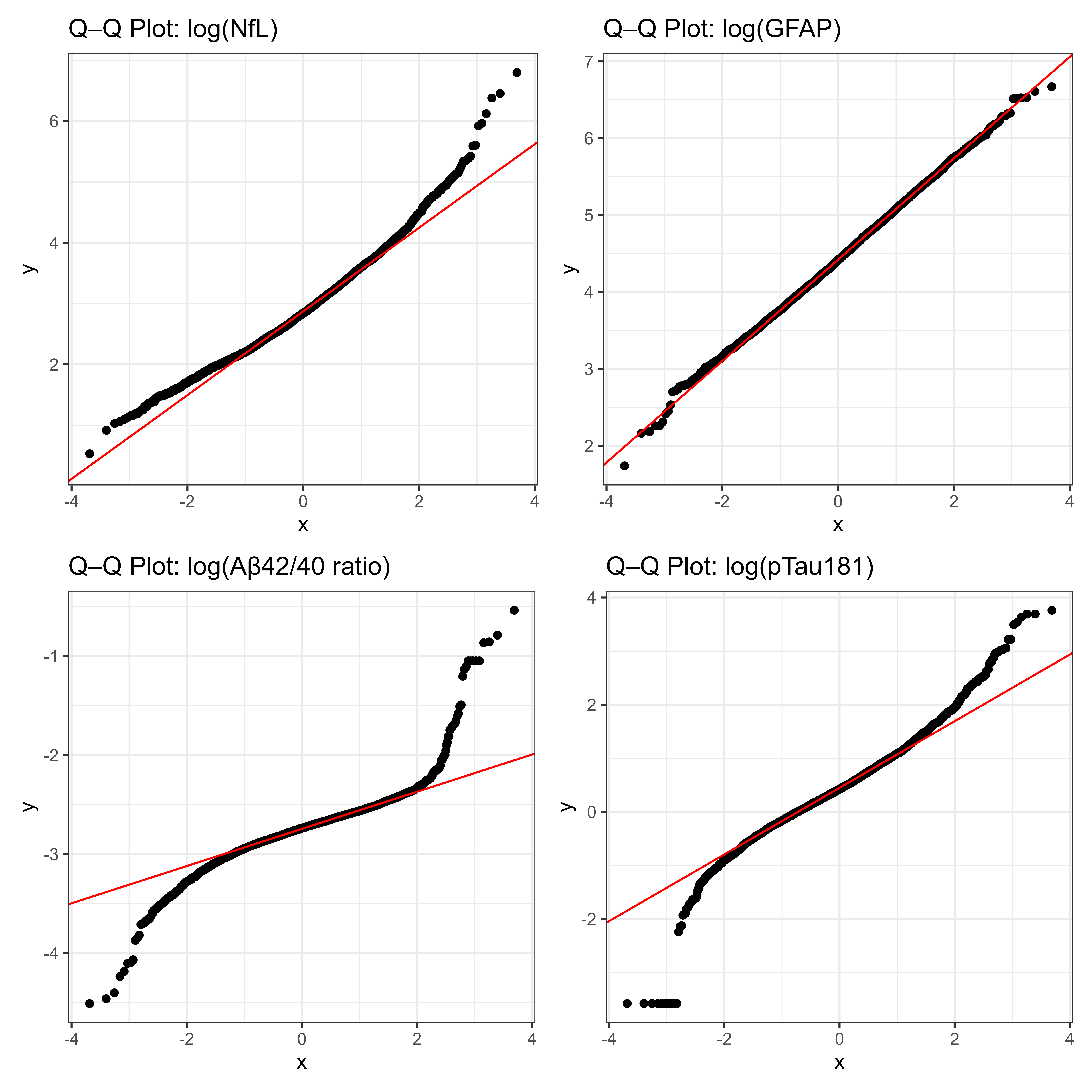

Supplement: Supplement 2 [file media-2.tif]

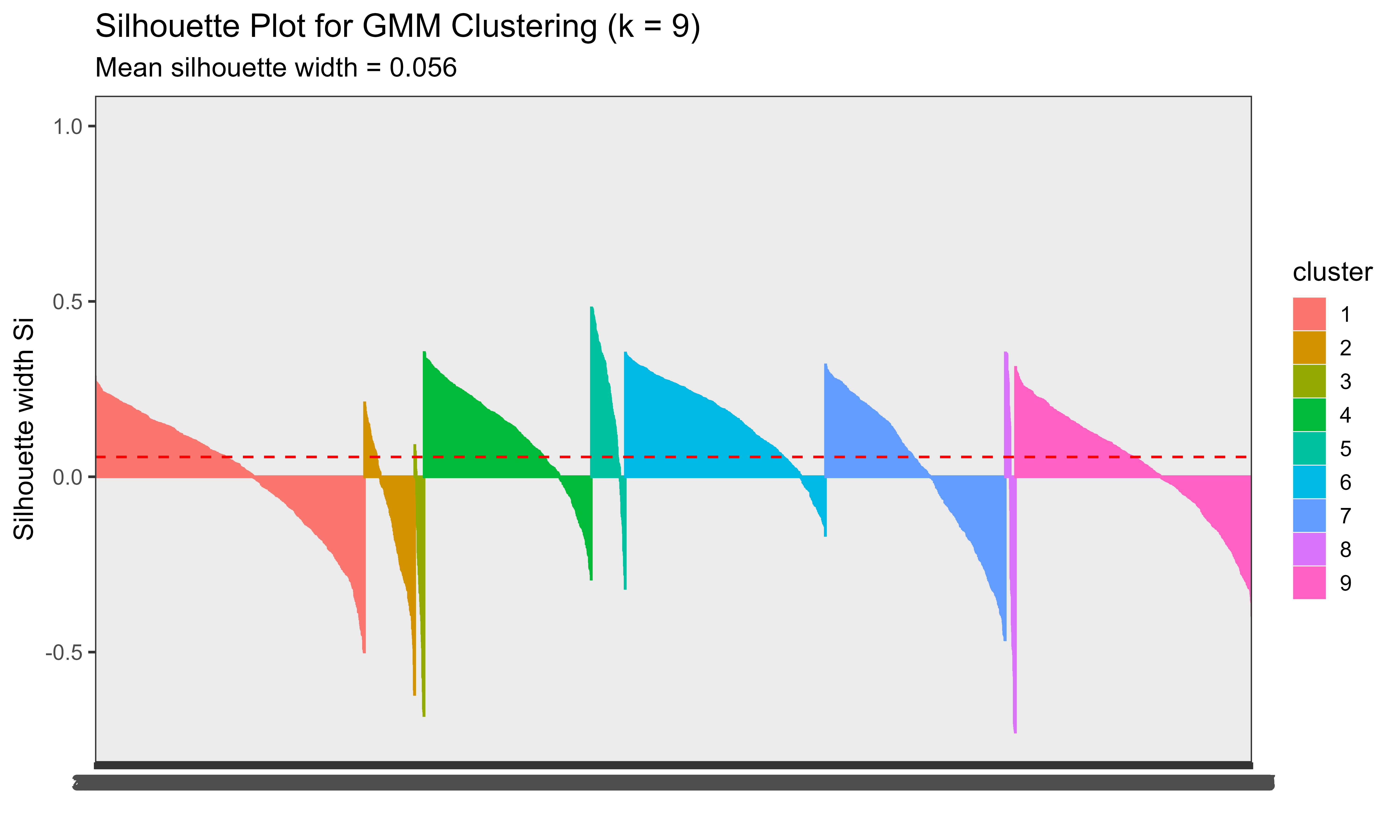

Supplement: Supplement 3 [file media-3.tif]

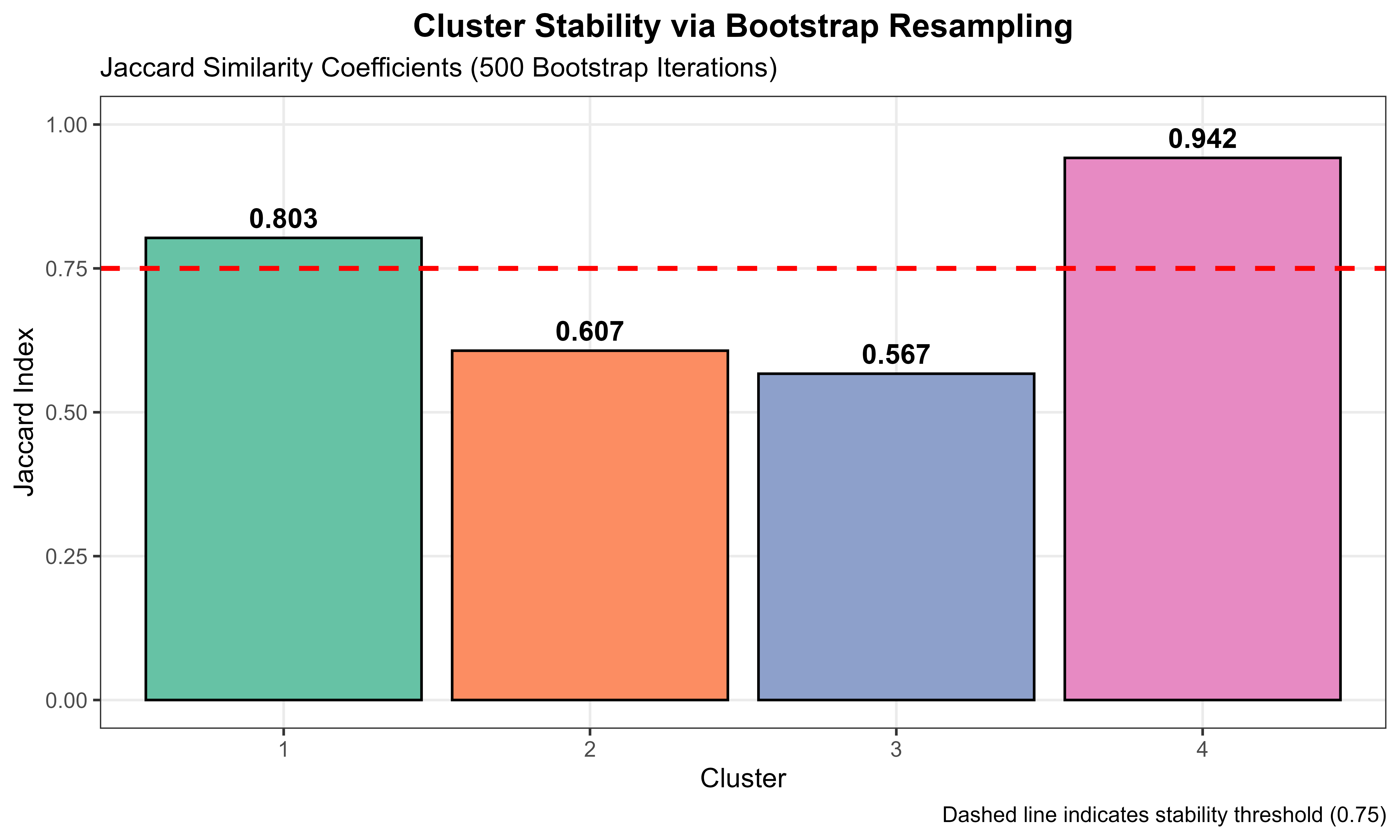

Supplement: Supplement 4 [file media-4.tif]

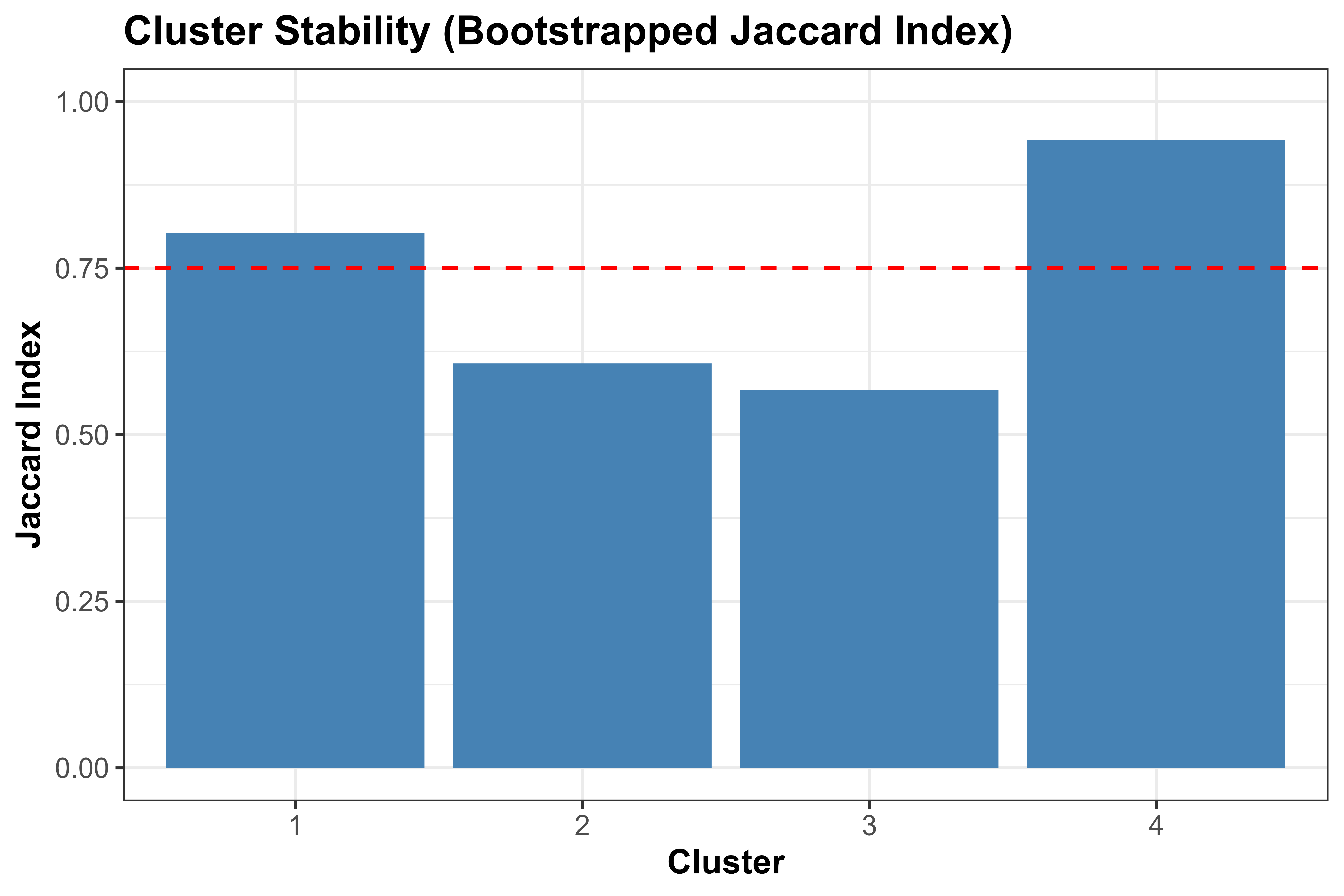

Supplement: Supplement 5 [file media-5.tif]

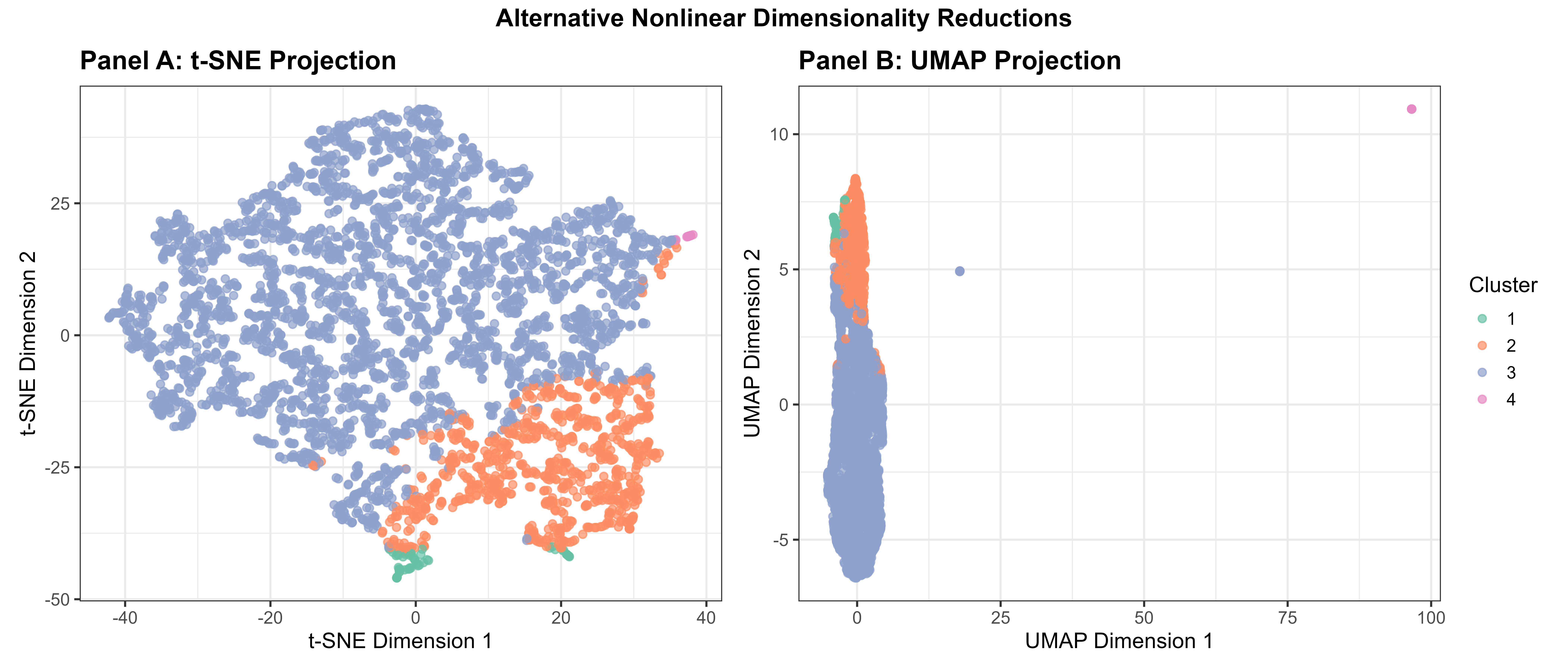

Supplement: Supplement 6 [file media-6.tif]

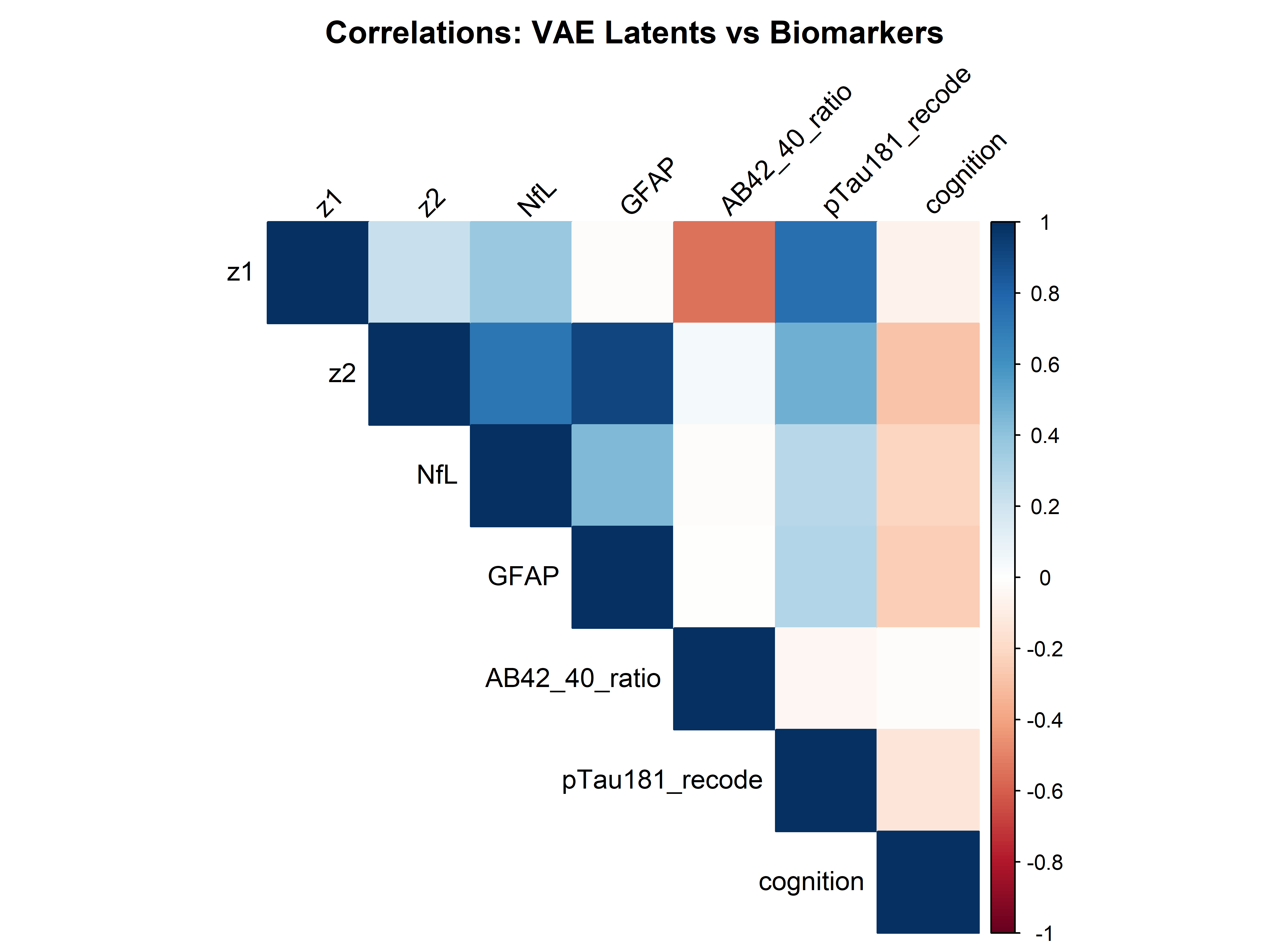

Supplement: Supplement 7 [file media-7.tif]

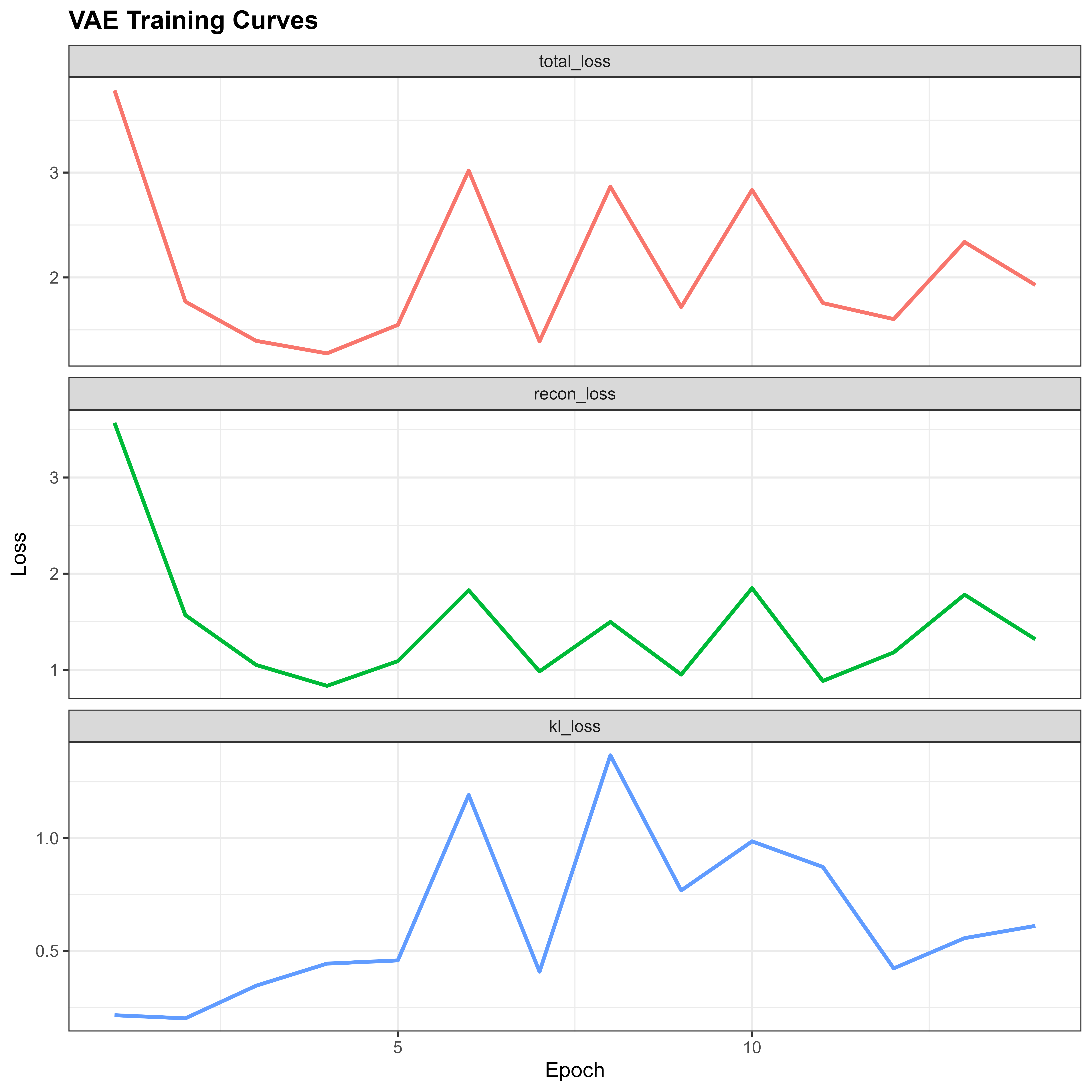

Supplement: Supplement 8 [file media-8.tif]

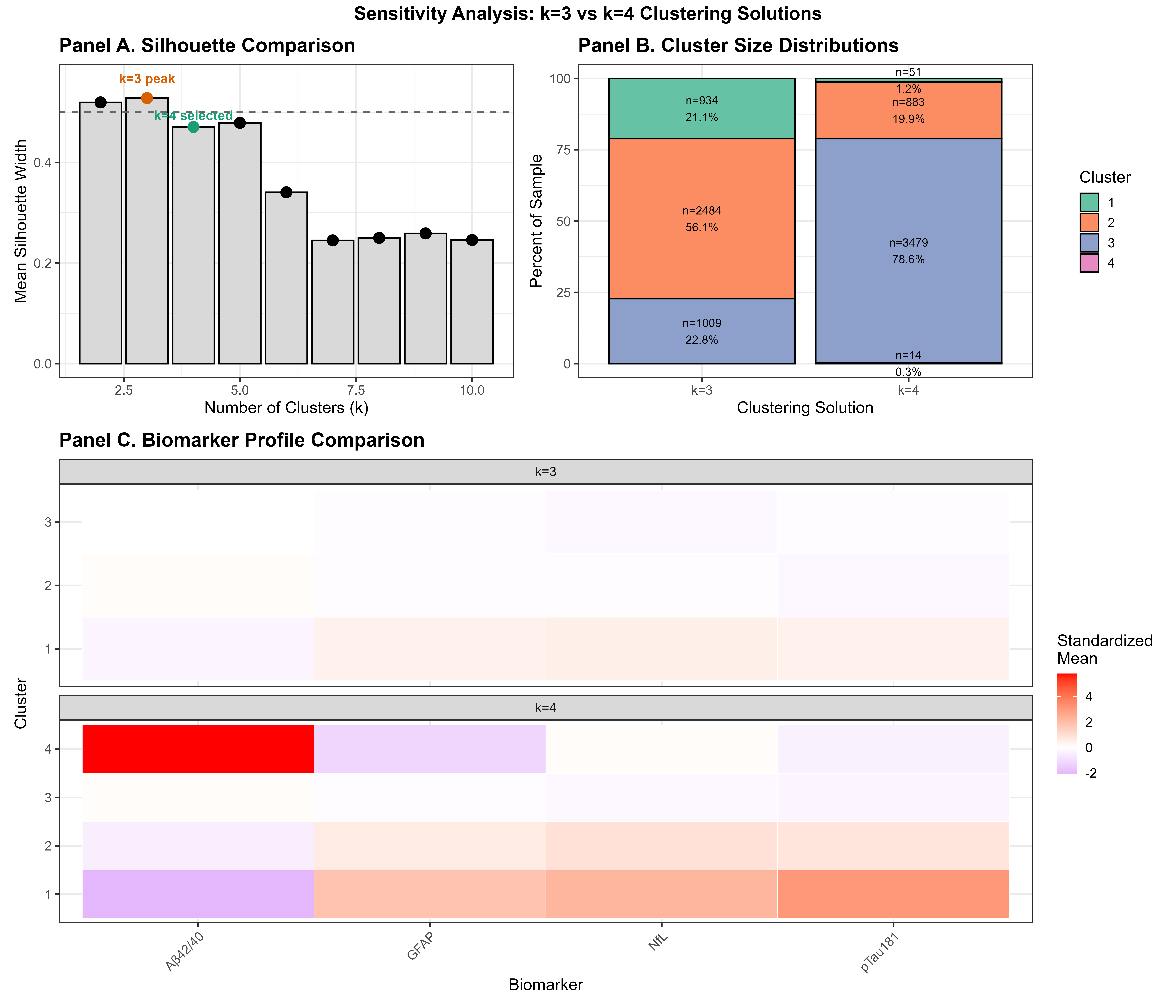

Supplement: Supplement 9 [file media-9.tif]

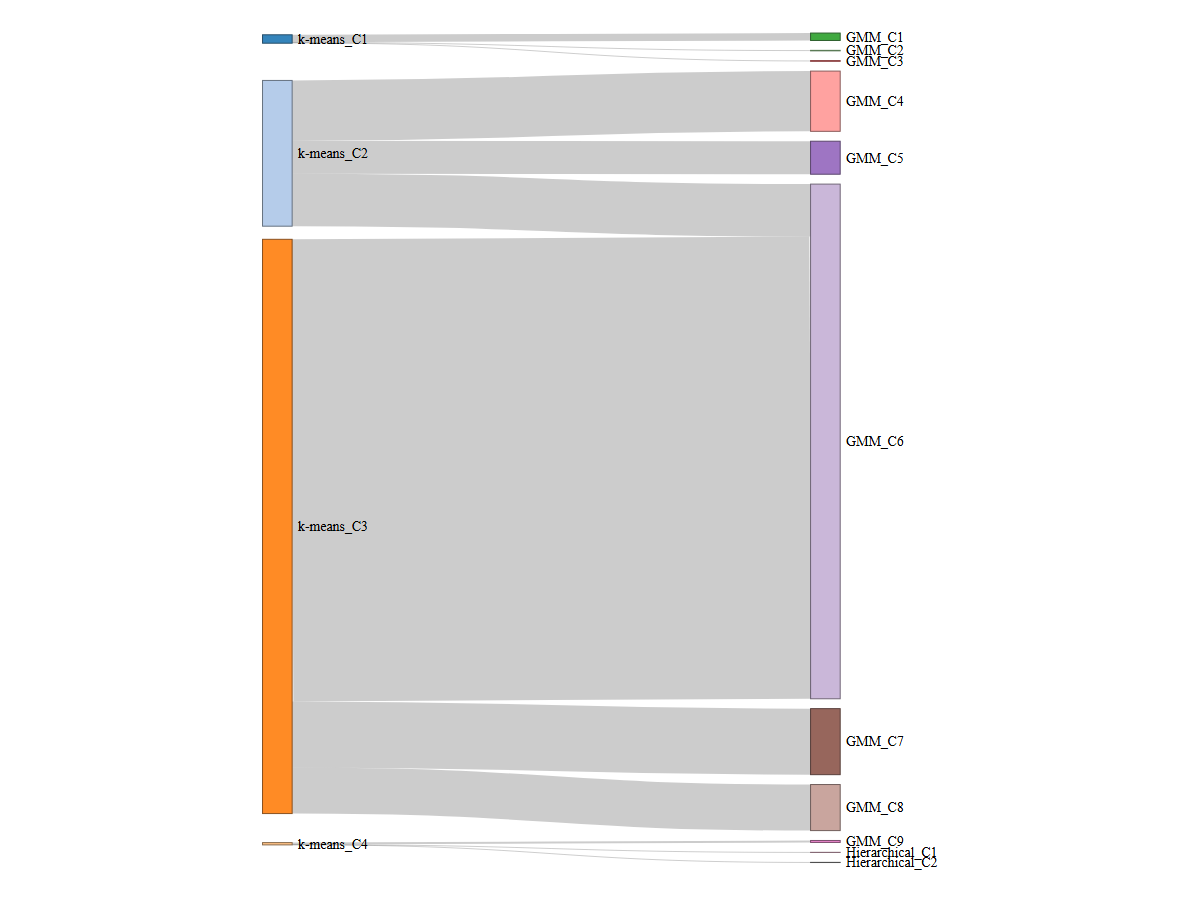

Supplement: Supplement 11 [file media-11.tif]
