# Supplementary material for: ATN Classification and Machine-Learned Plasma Biomarker Phenotypes Reveal Distinct Alzheimer’s Pathology in a Population-Based Cohort
Source: medRxiv. 2026 Jan 30:2026.01.02.26343331. Originally published 2026 Jan 6. Preprint. [Version 3] doi: 10.64898/2026.01.02.26343331 (PMC12803393; doi:10.64898/2026.01.02.26343331)
Supplement: Supplementary file 1 [file NIHPP2026.01.02.26343331v3-supplement-2.pdf]

A

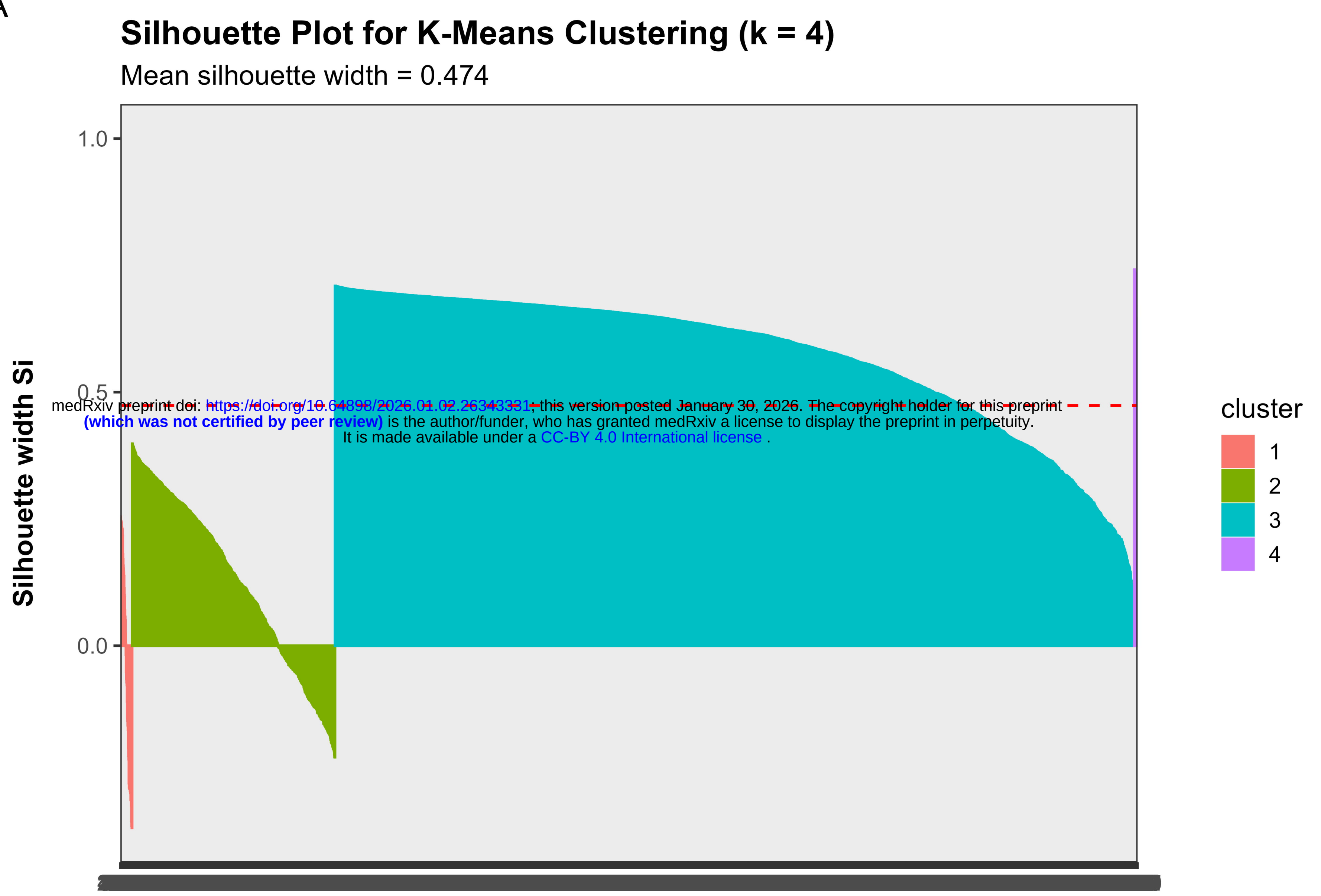

medRxiv preprint doi: https://doi.org/10.64898/2026.01.02.26343331; this version posted January 30, 2026. The copyright holder for this preprint (which was not certified by peer review) is the author/funder, who has granted medRxiv a license to display the preprint in perpetuity.
It is made available under a CC-BY 4.0 International license .

B

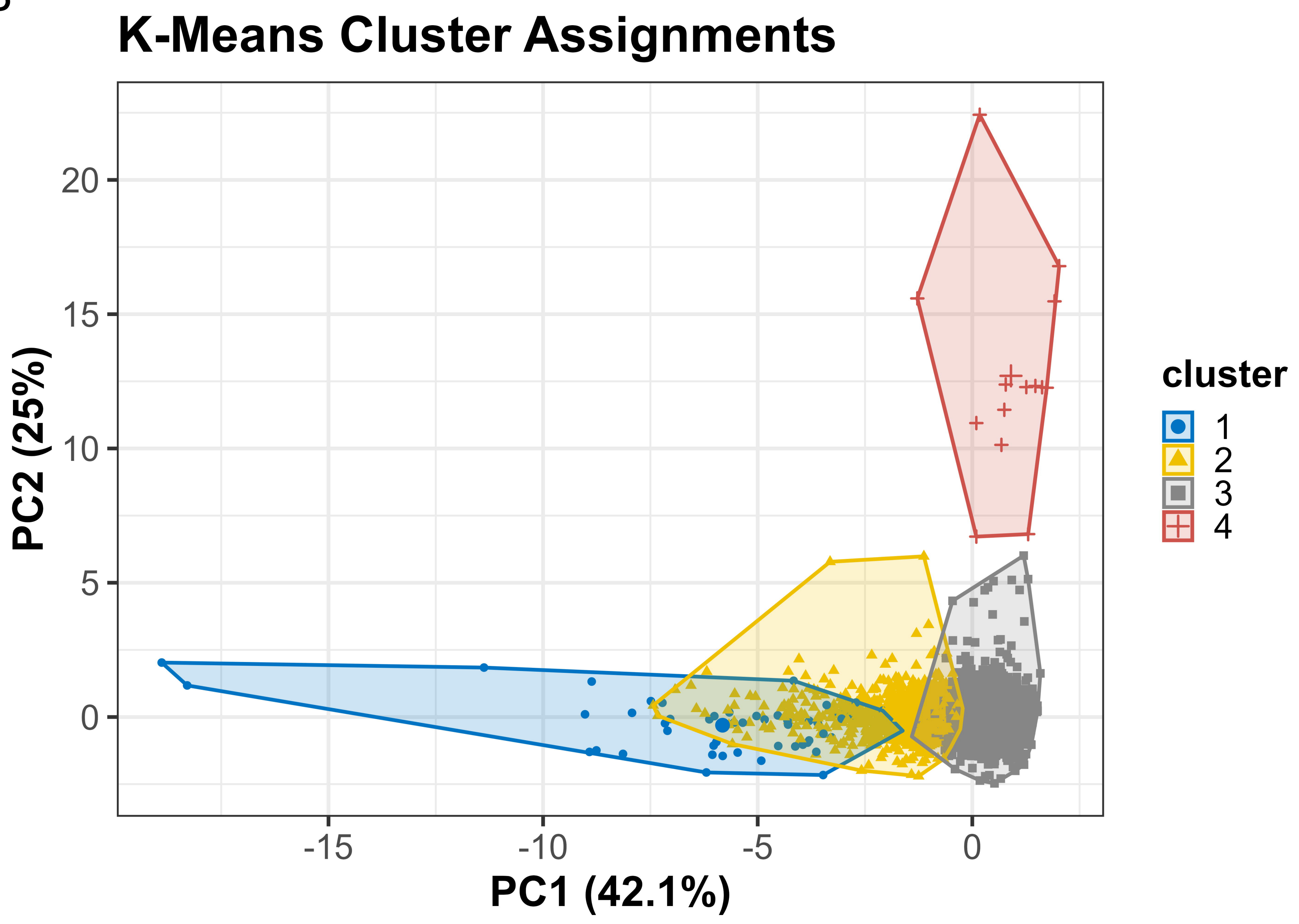

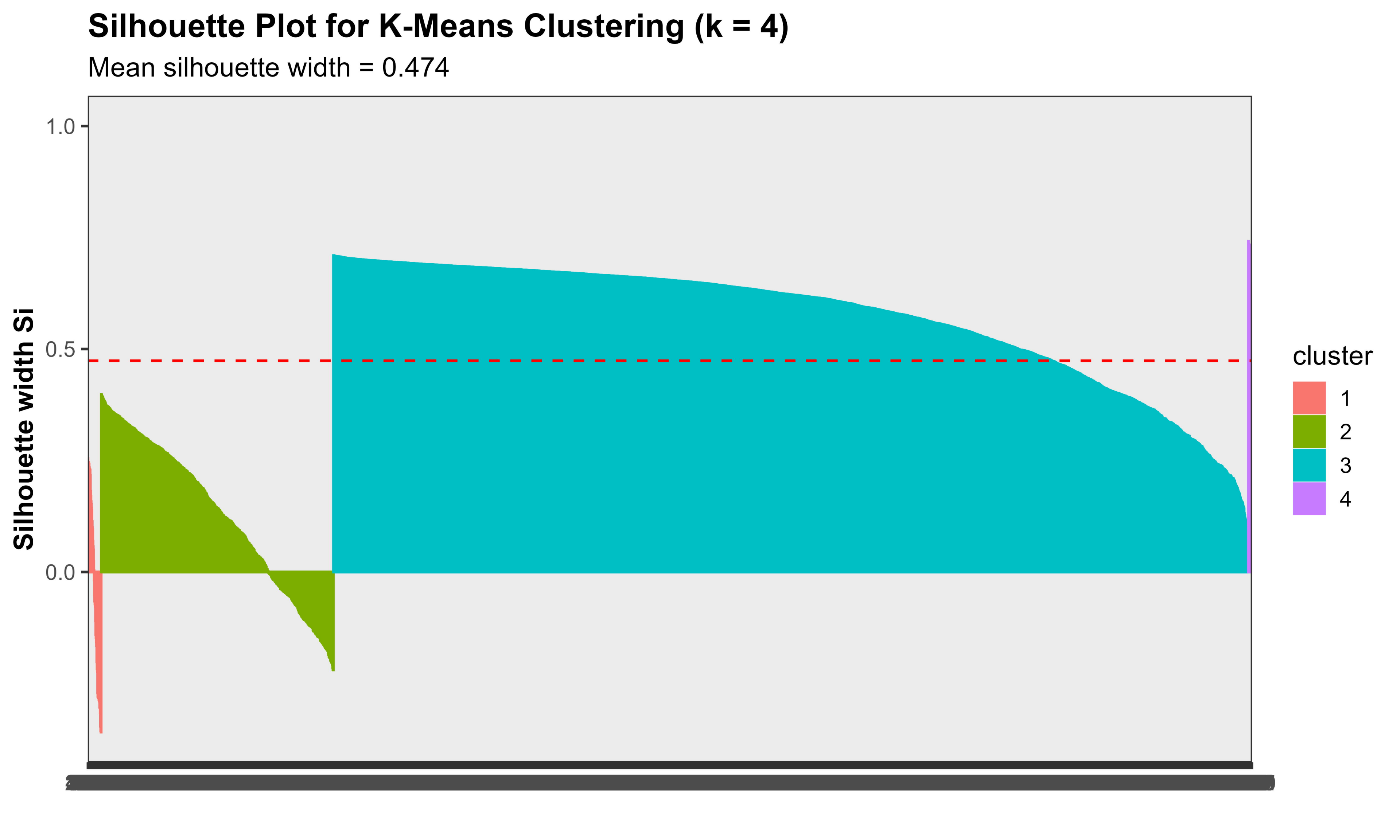
Silhouette Plot for K-Means Clustering (k = 4)
Mean silhouette width = 0.474
1.0
0.5
0.0
Silhouette width Si
cluster
1
2
3
4

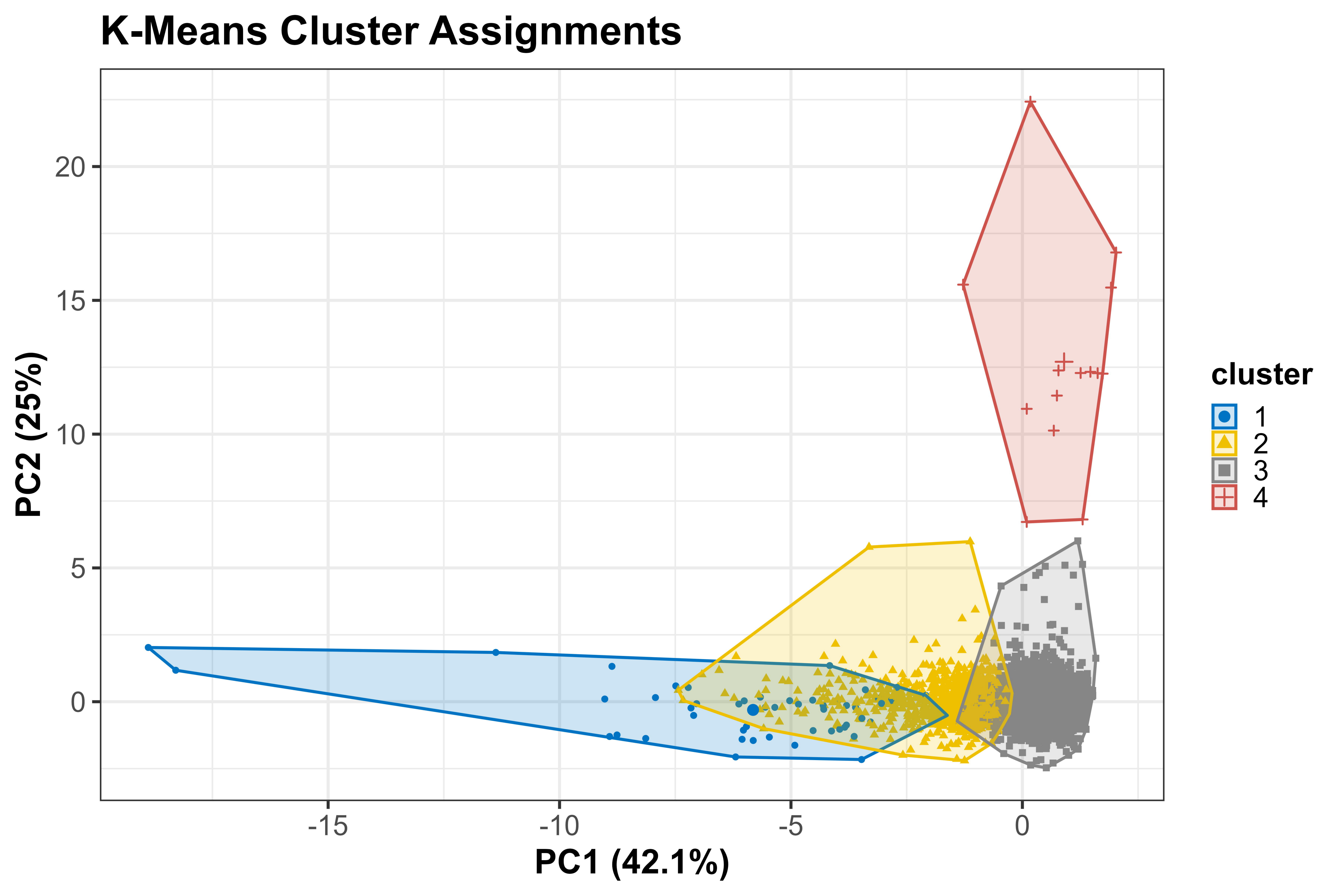
K-Means Cluster Assignments
PC2 (25%)
PC1 (42.1%)
20
15
10
5
0
-15
-10
-5
0
cluster
1
2
3
4

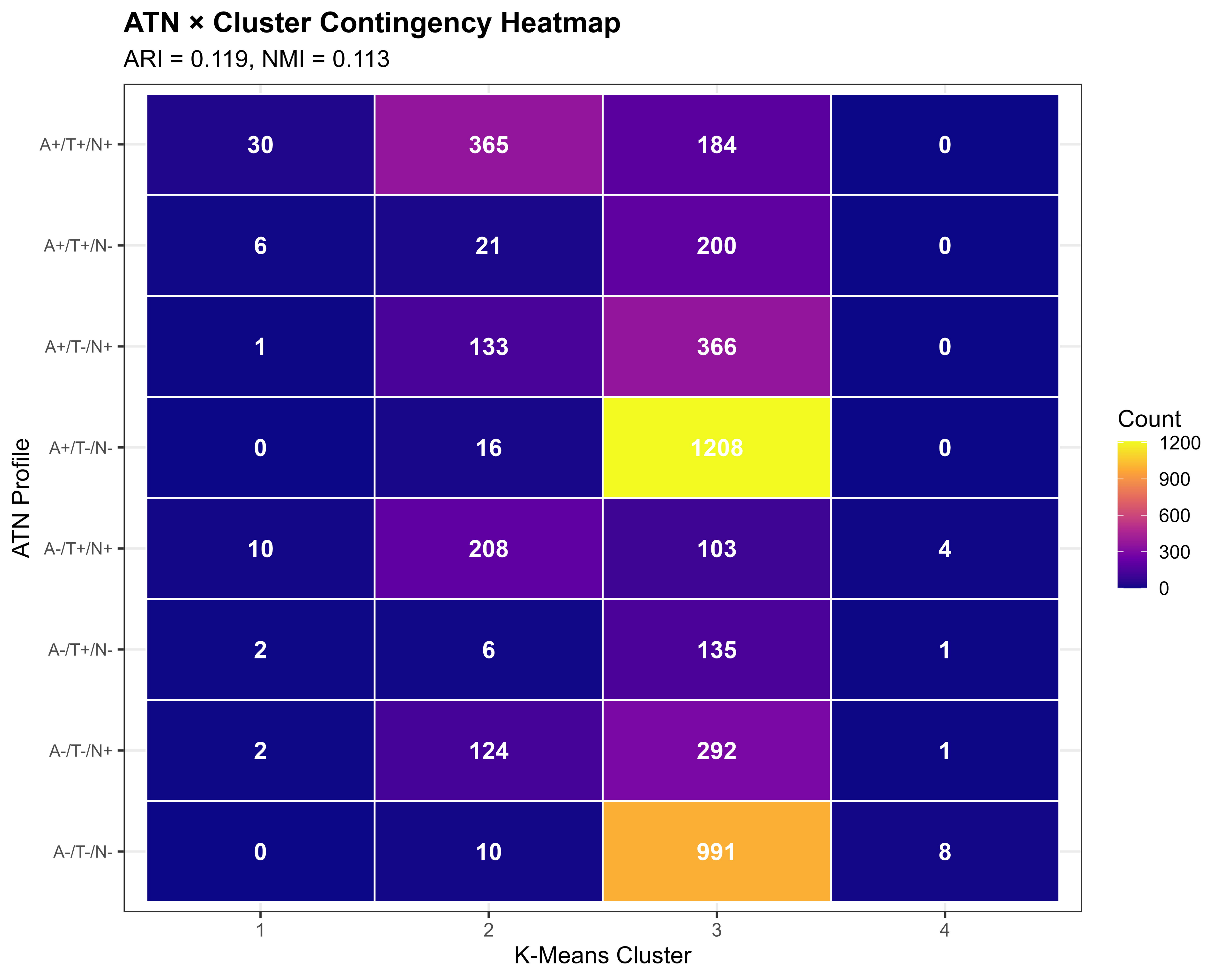
ATN × Cluster Contingency Heatmap
ARI = 0.119, NMI = 0.113
ATN Profile
A+/T+/N+
A+/T+/N-
A+/T-/N+
A+/T-/N-
A-/T+/N+
A-/T+/N-
A-/T-/N+
A-/T-/N-
30
365
184
0
6
21
200
0
1
133
366
0
0
16
1208
0
10
208
103
4
2
6
135
1
2
124
292
1
0
10
991
8
1
2
3
4
K-Means Cluster
Count
1200
900
600
300
0

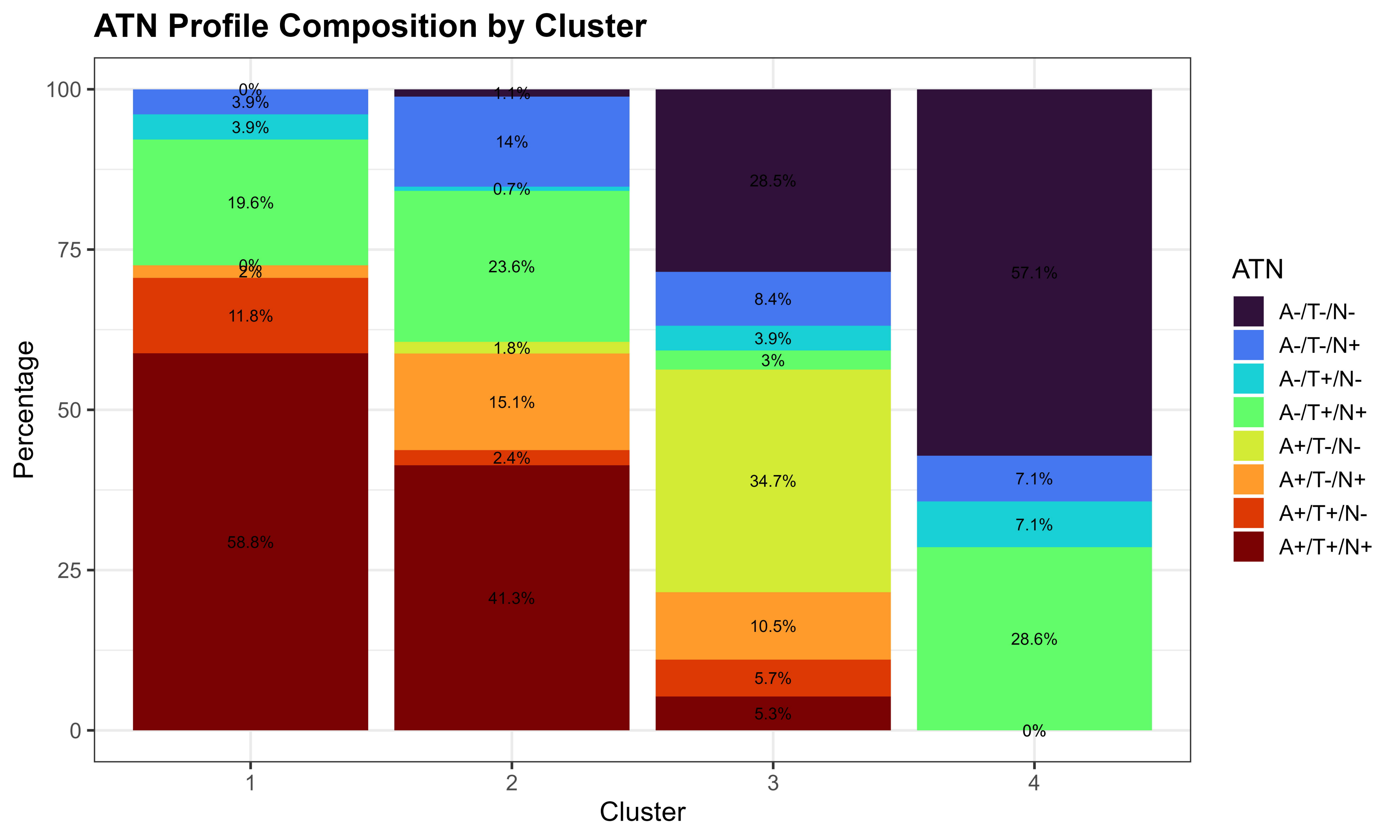

ATN Profile Composition by Cluster
Percentage
100
75
50
25
0
Cluster
1
2
3
4
ATN
A-/T-/N-
A-/T-/N+
A-/T+/N-
A-/T+/N+
A+/T-/N-
A+/T-/N+
A+/T+/N-
A+/T+/N+
0%
3.9%
3.9%
19.6%
0%
2%
11.8%
58.8%
1.1%
14%
0.7%
23.6%
1.8%
15.1%
2.4%
41.3%
28.5%
8.4%
3.9%
3%
34.7%
10.5%
5.7%
5.3%
57.1%
7.1%
7.1%
28.6%
0%

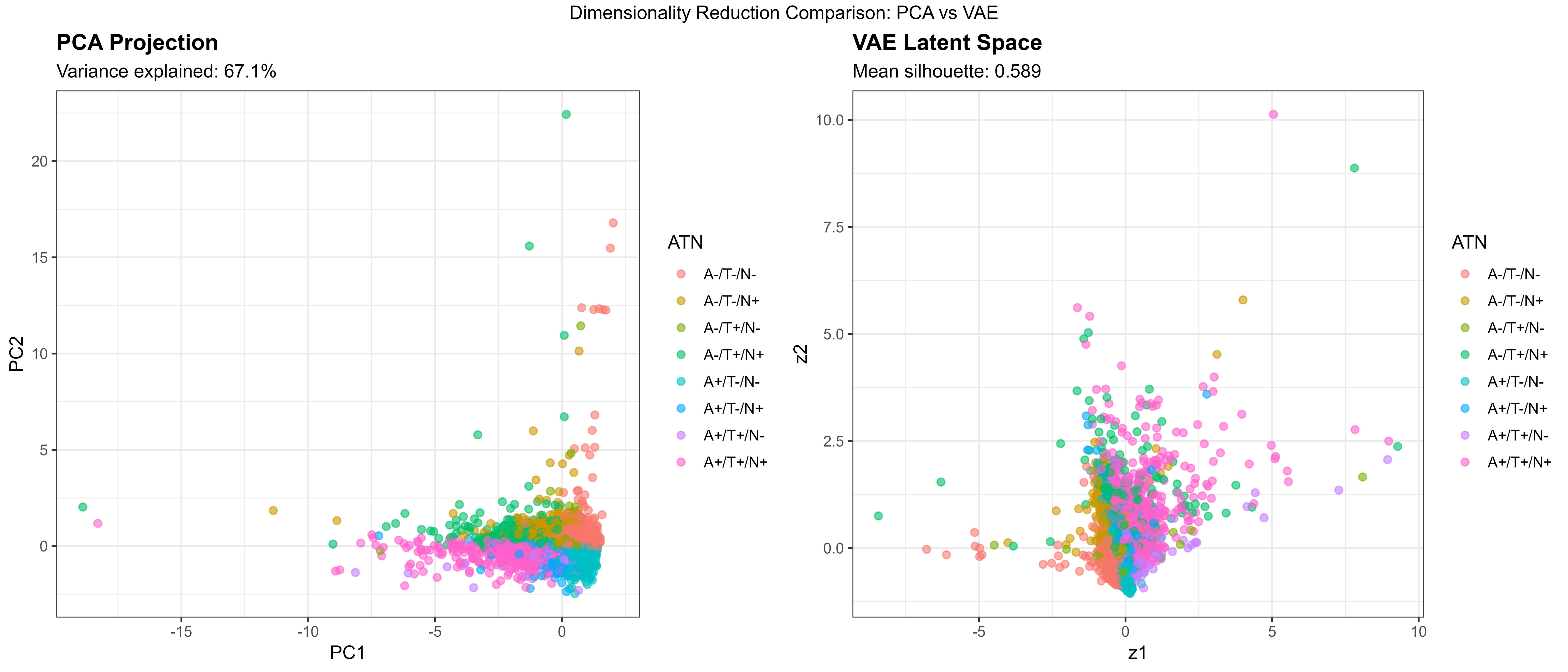
Dimensionality Reduction Comparison: PCA vs VAE
PCA Projection
Variance explained: 67.1%
PC1
PC2
-15
-10
-5
0
0
5
10
15
20
ATN
A-/T-/N-
A-/T-/N+
A-/T+/N-
A-/T+/N+
A+/T-/N-
A+/T-/N+
A+/T+/N-
A+/T+/N+
VAE Latent Space
Mean silhouette: 0.589
z1
z2
-5
0
5
10
0.0
2.5
5.0
7.5
10.0
ATN
A-/T-/N-
A-/T-/N+
A-/T+/N-
A-/T+/N+
A+/T-/N-
A+/T-/N+
A+/T+/N-
A+/T+/N+

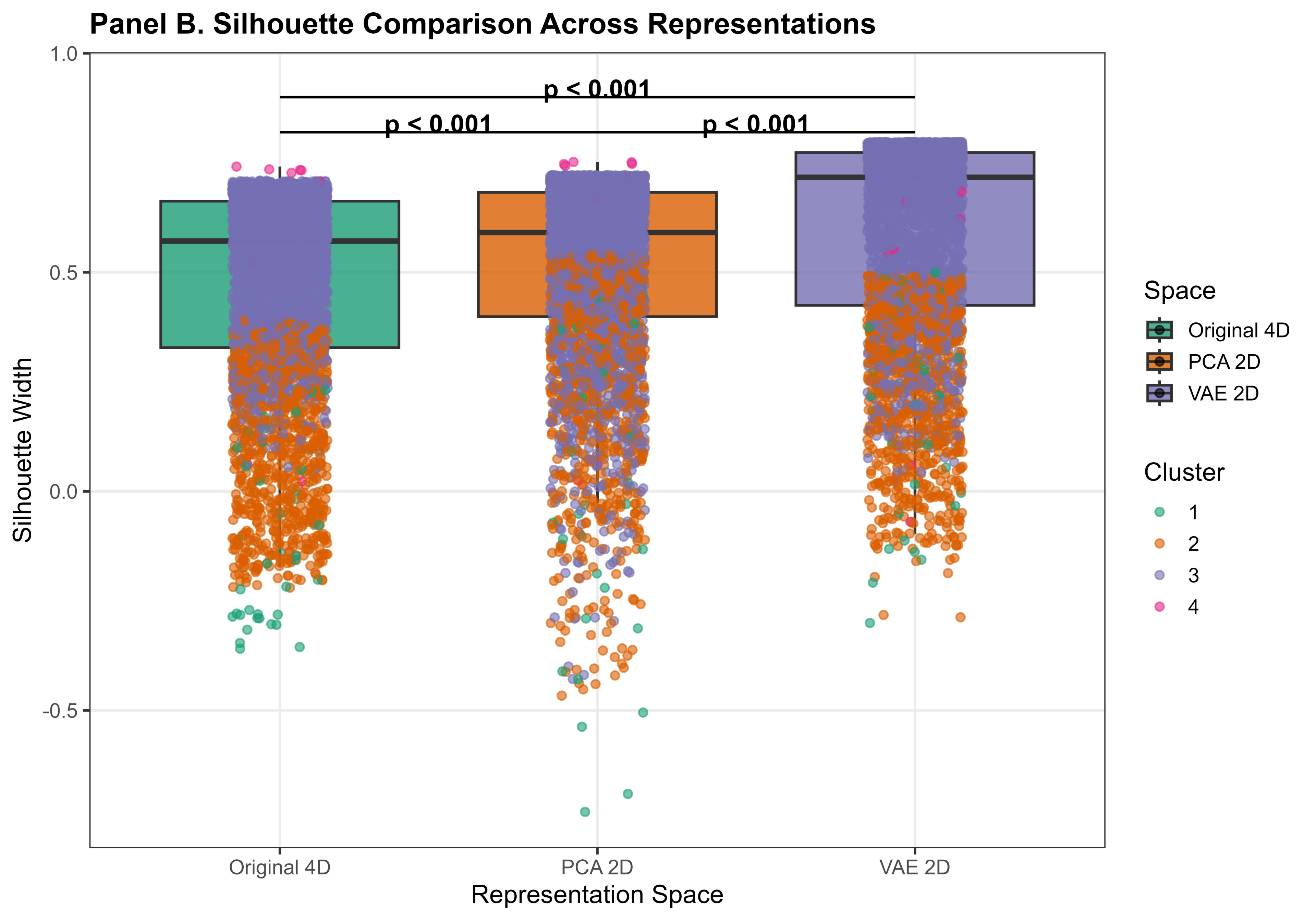

Panel B. Silhouette Comparison Across Representations
p < 0.001
p < 0.001
p < 0.001
1.0
0.5
0.0
-0.5
Silhouette Width
Original 4D
PCA 2D
VAE 2D
Representation Space
Space
Original 4D
PCA 2D
VAE 2D
Cluster
1
2
3
4

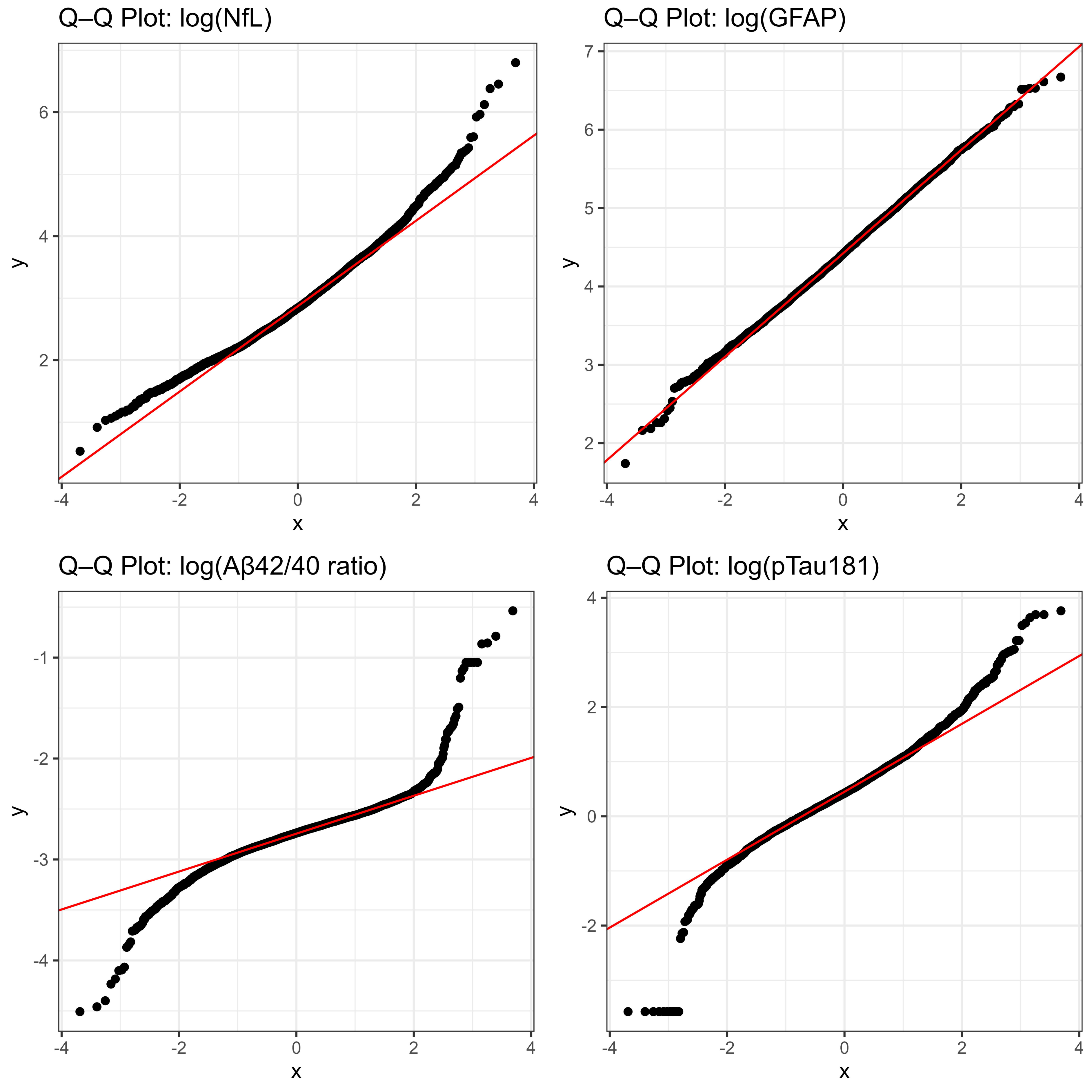

Q–Q Plot: log(NfL)
Q–Q Plot: log(GFAP)
Q–Q Plot: log(Aβ42/40 ratio)
Q–Q Plot: log(pTau181)
x
y

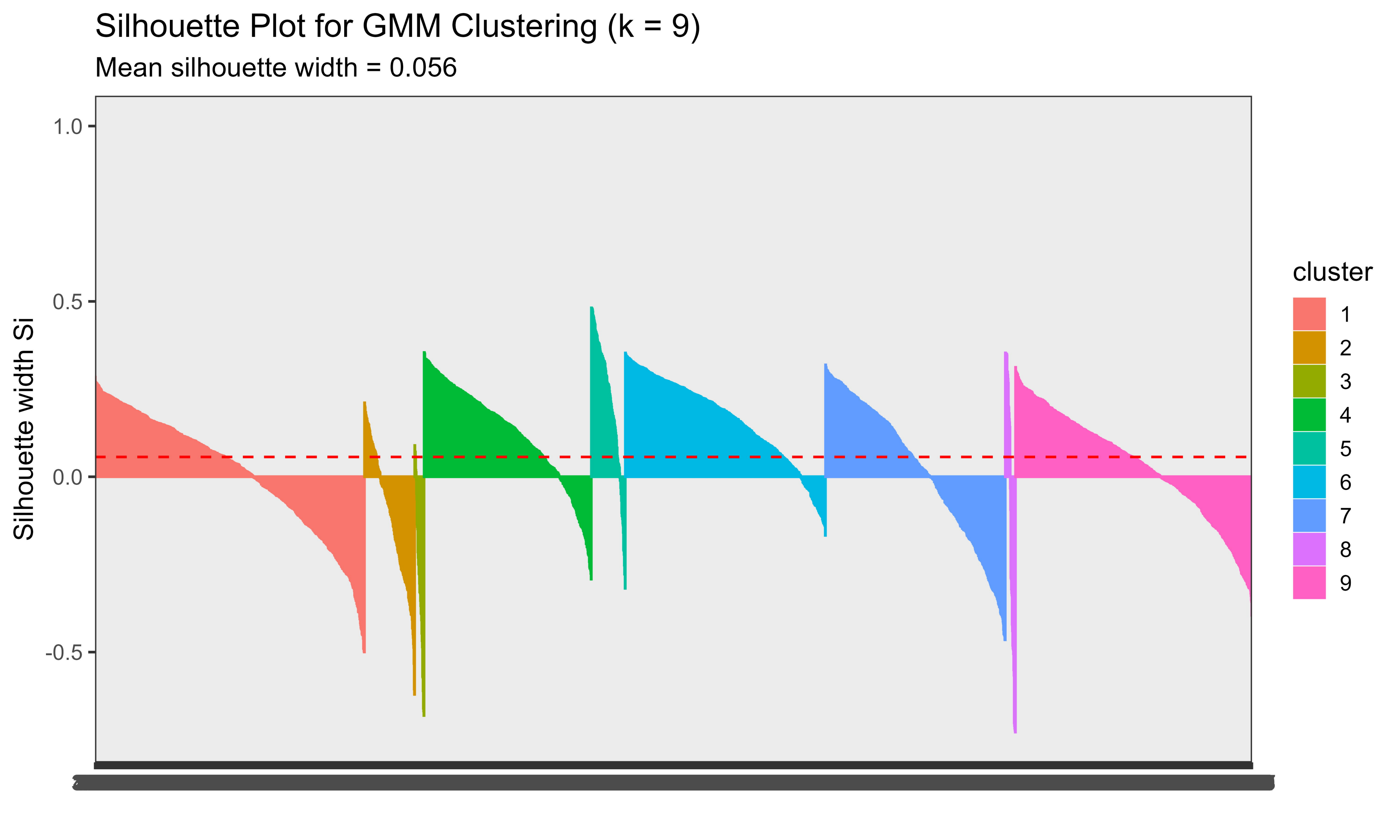

Silhouette Plot for GMM Clustering (k = 9)
Mean silhouette width = 0.056
Silhouette width Si
1.0
0.5
0.0
-0.5
cluster
1
2
3
4
5
6
7
8
9

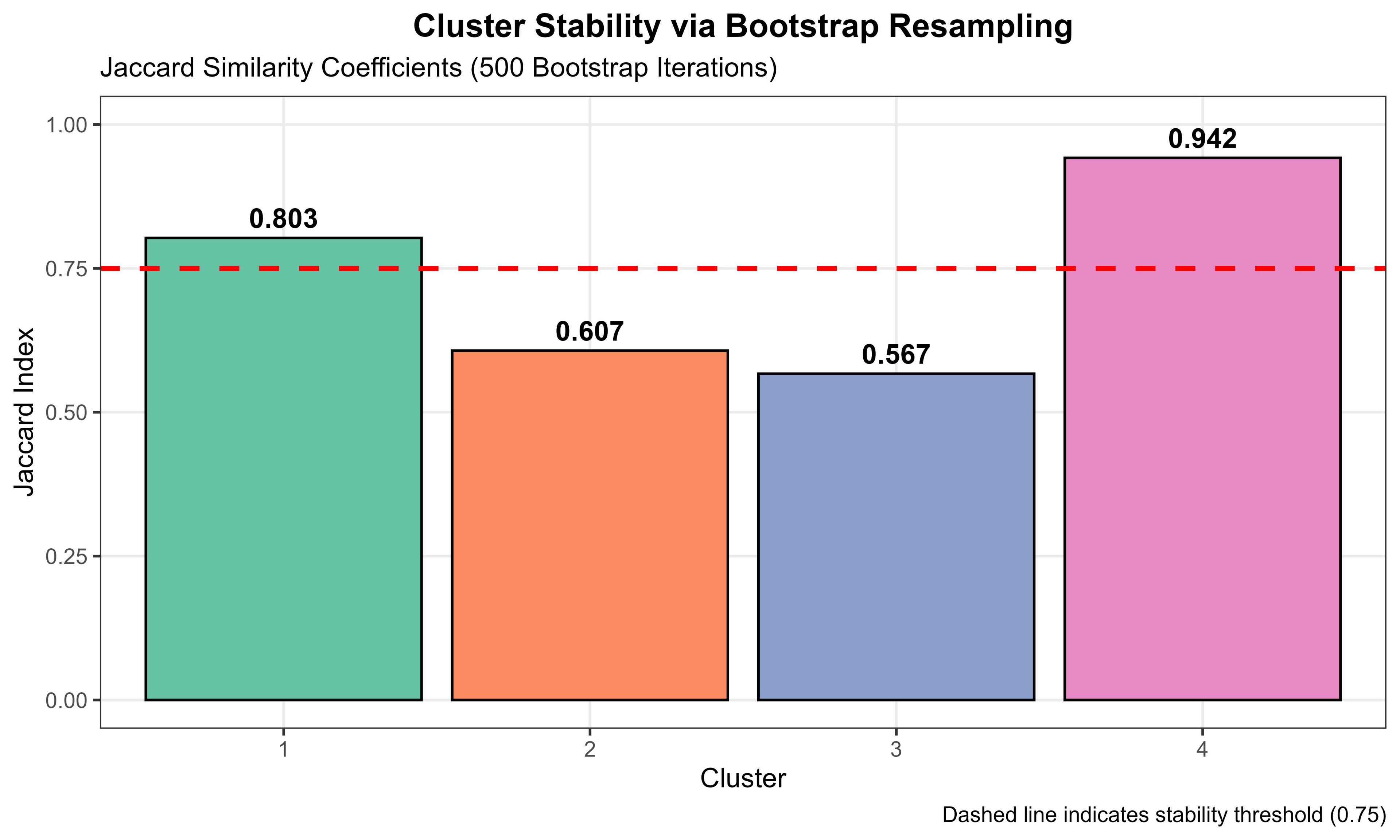
Cluster Stability via Bootstrap Resampling
Jaccard Similarity Coefficients (500 Bootstrap Iterations)
1.00
0.75
0.50
0.25
0.00
Jaccard Index
0.803
0.607
0.567
0.942
1
2
3
4
Cluster
Dashed line indicates stability threshold (0.75)

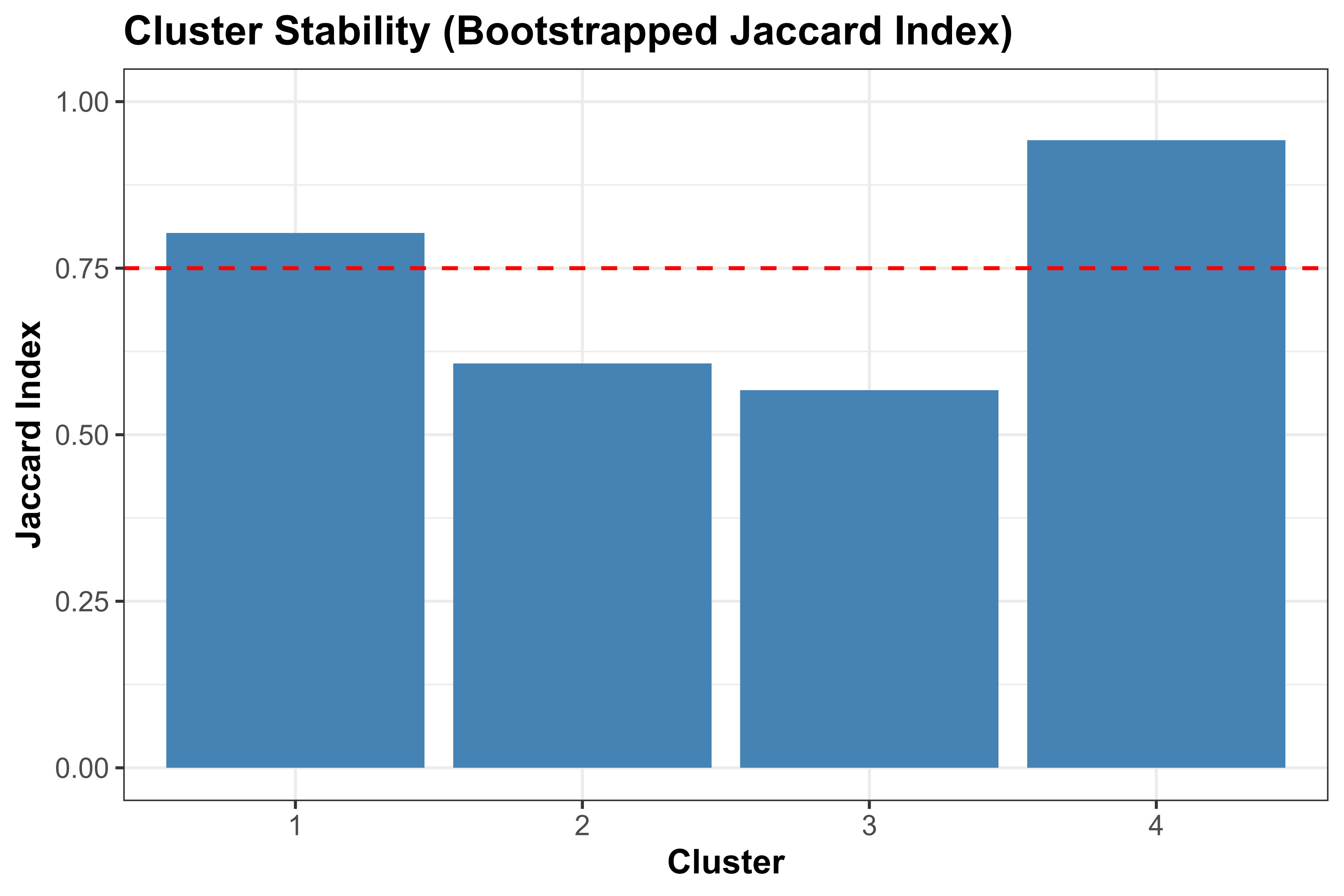
Cluster Stability (Bootstrapped Jaccard Index)
Jaccard Index
1.00
0.75
0.50
0.25
0.00
1
2
3
4
Cluster

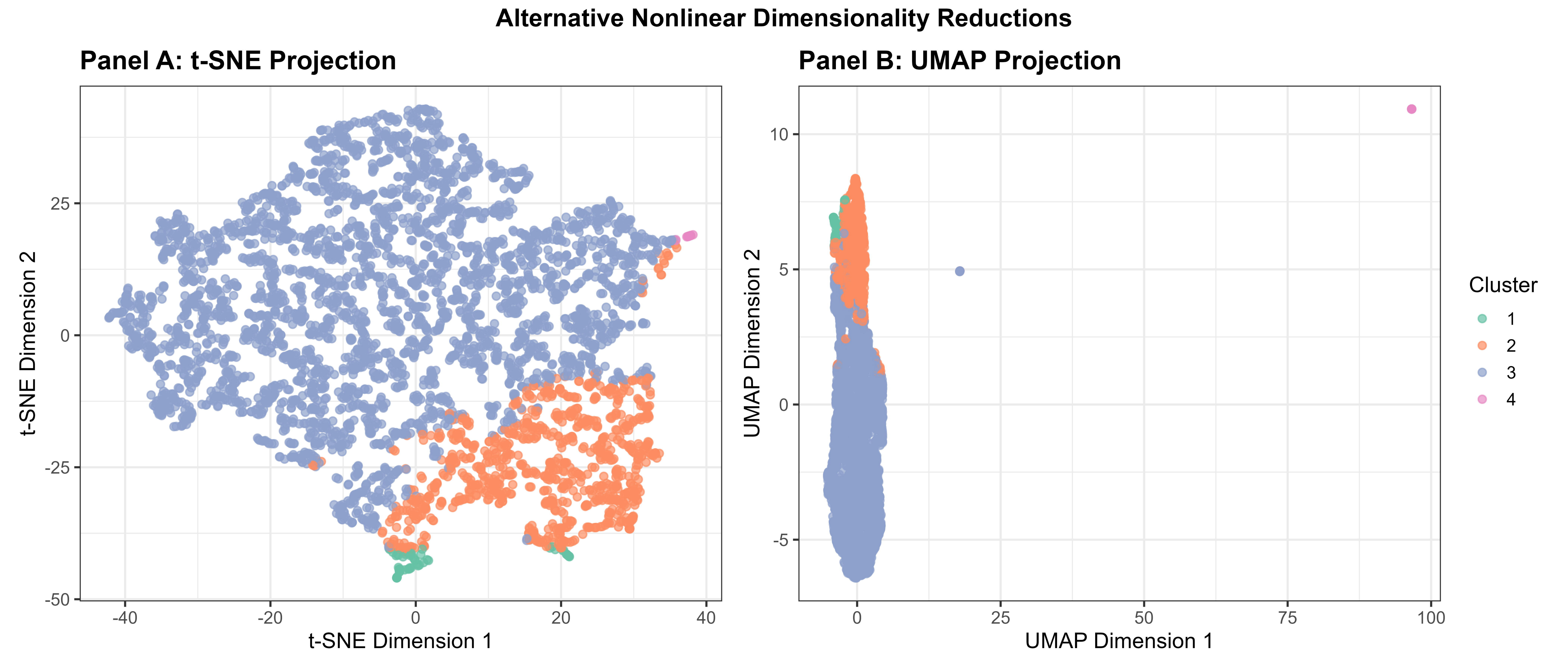

Alternative Nonlinear Dimensionality Reductions
Panel A: t-SNE Projection
25
0
-25
-50
-40
-20
0
20
40
t-SNE Dimension 1
t-SNE Dimension 2
Panel B: UMAP Projection
10
5
0
-5
0
25
50
75
100
UMAP Dimension 1
UMAP Dimension 2
Cluster
1
2
3
4

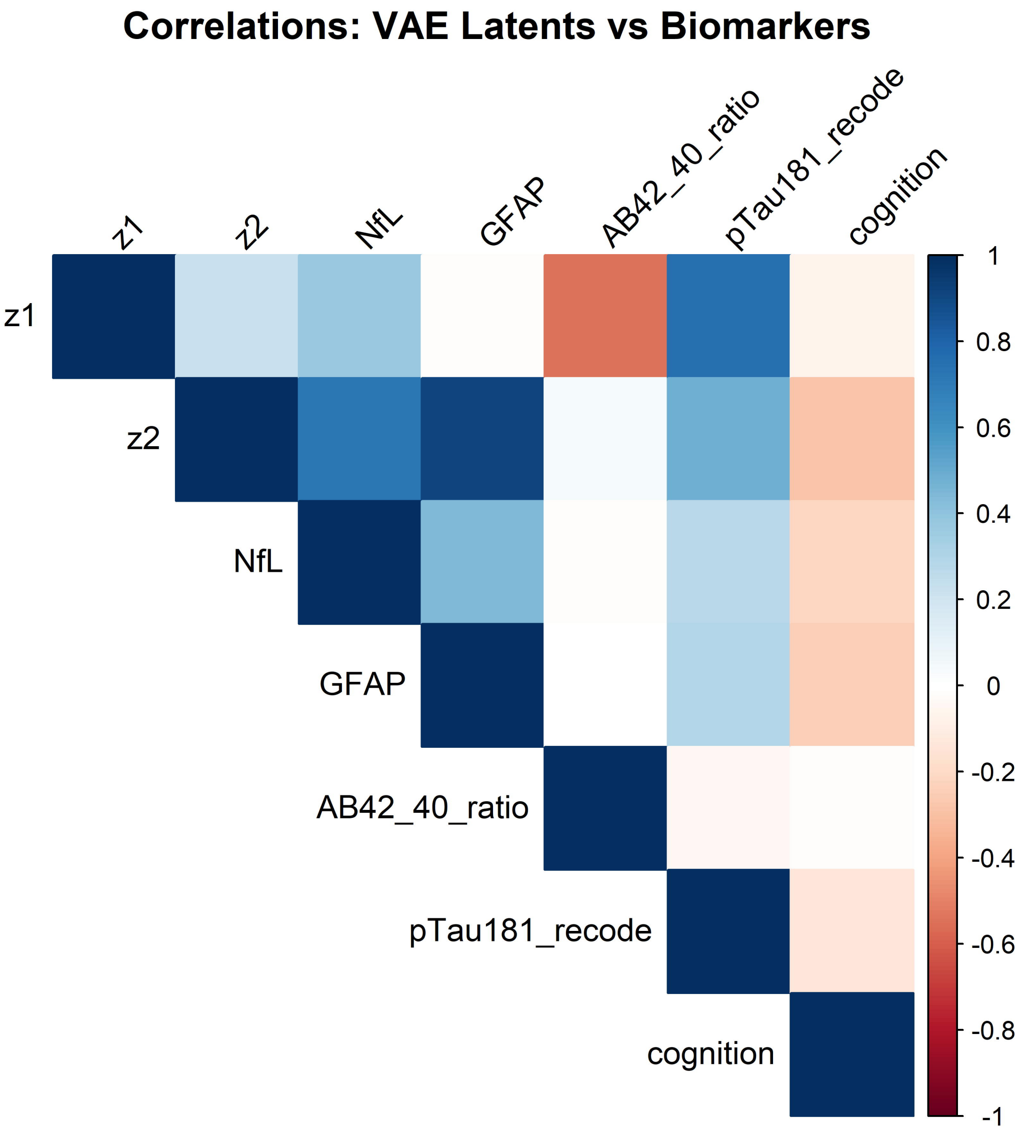
Correlations: VAE Latents vs Biomarkers
z1
z2
NfL
GFAP
AB42_40_ratio
pTau181_recode
cognition
1
0.8
0.6
0.4
0.2
0
-0.2
-0.4
-0.6
-0.8
-1

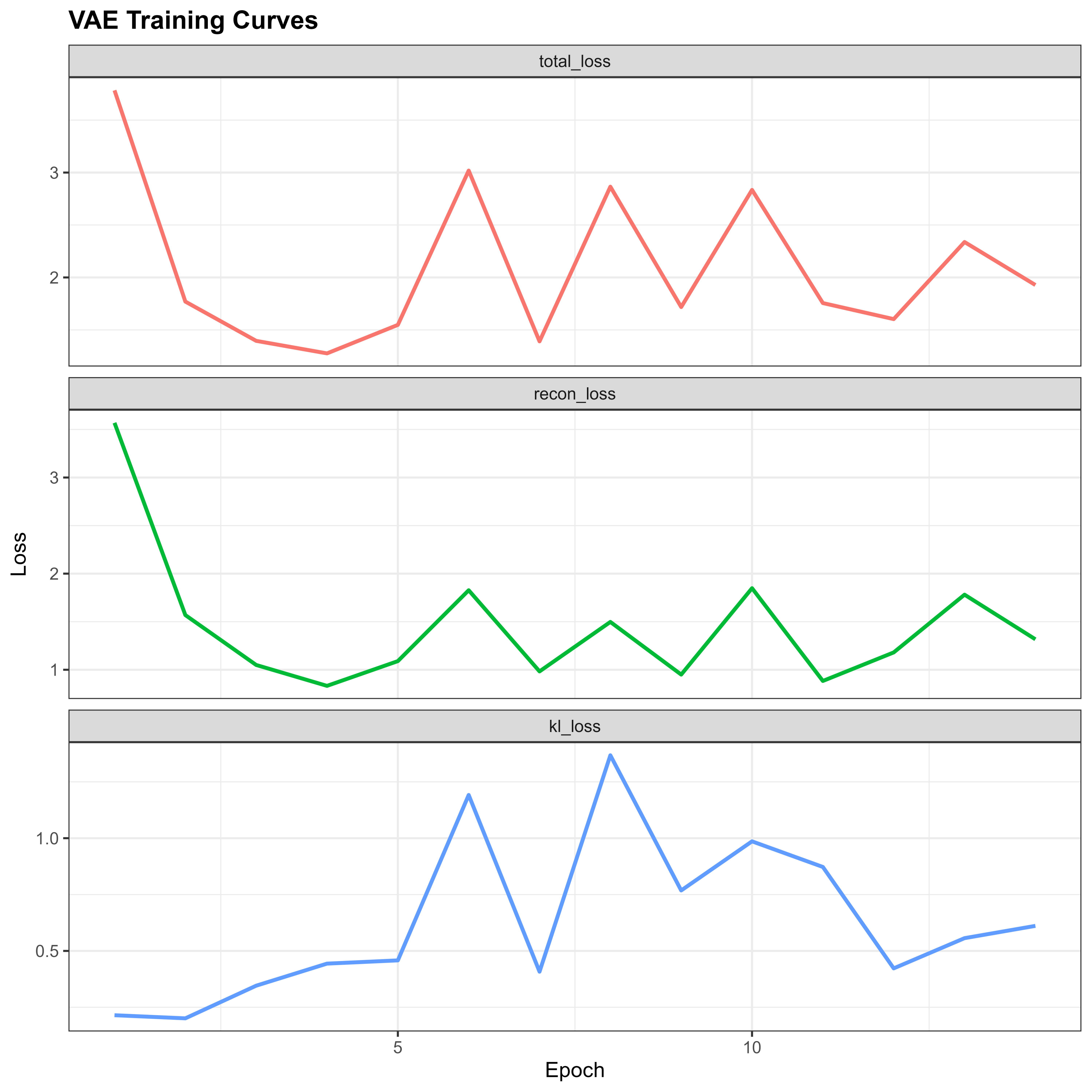

VAE Training Curves
total_loss
recon_loss
kl_loss
Loss
Epoch
3
2
3
2
1
1.0
0.5
5
10

medRxiv preprint doi: https://doi.org/10.64898/2026.01.02.26343331; this version posted January 30, 2026. The copyright holder for this preprint (which was not certified by peer review) is the author/funder, who has granted medRxiv a license to display the preprint in perpetuity.
It is made available under a CC-BY 4.0 International license.

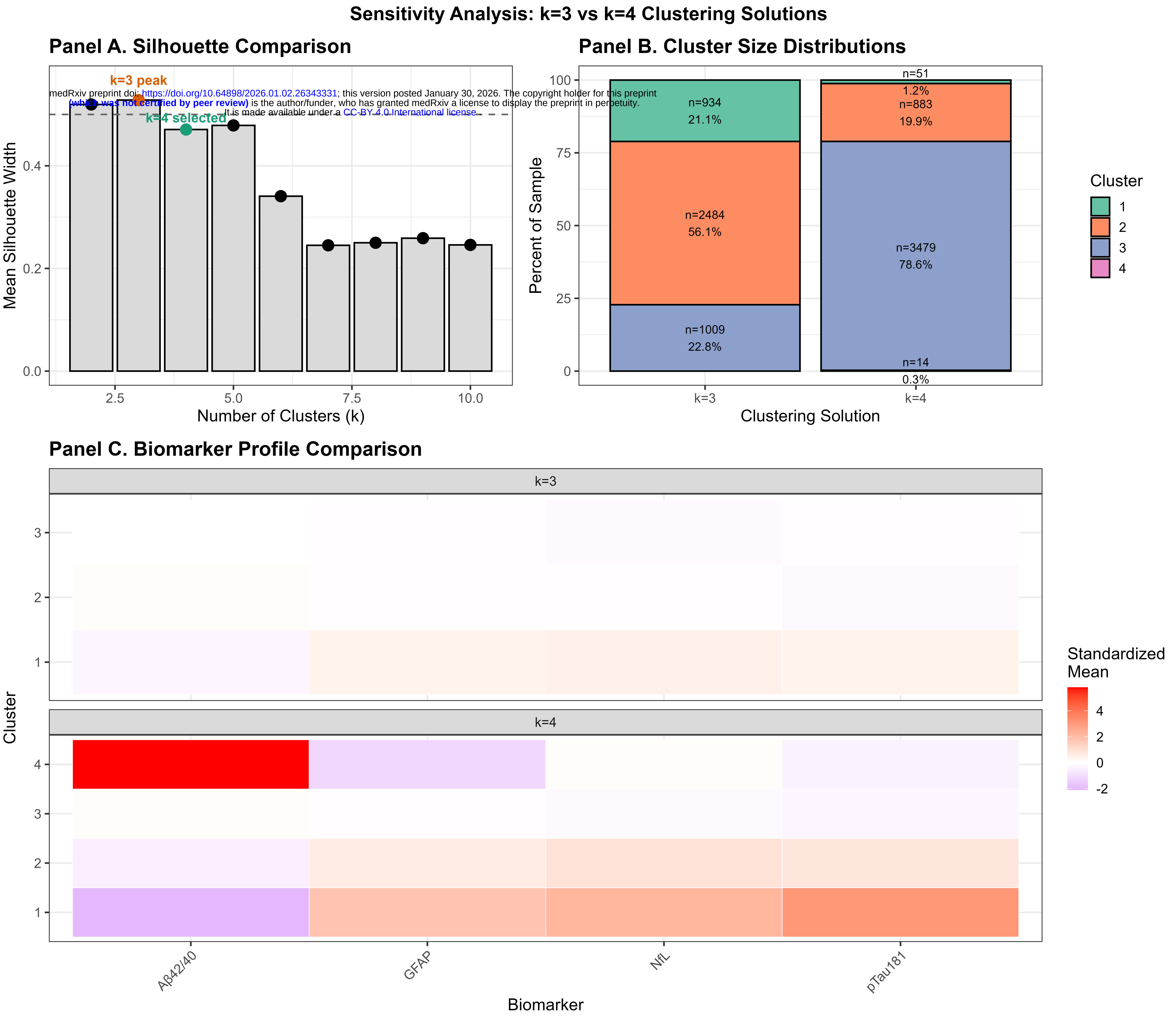

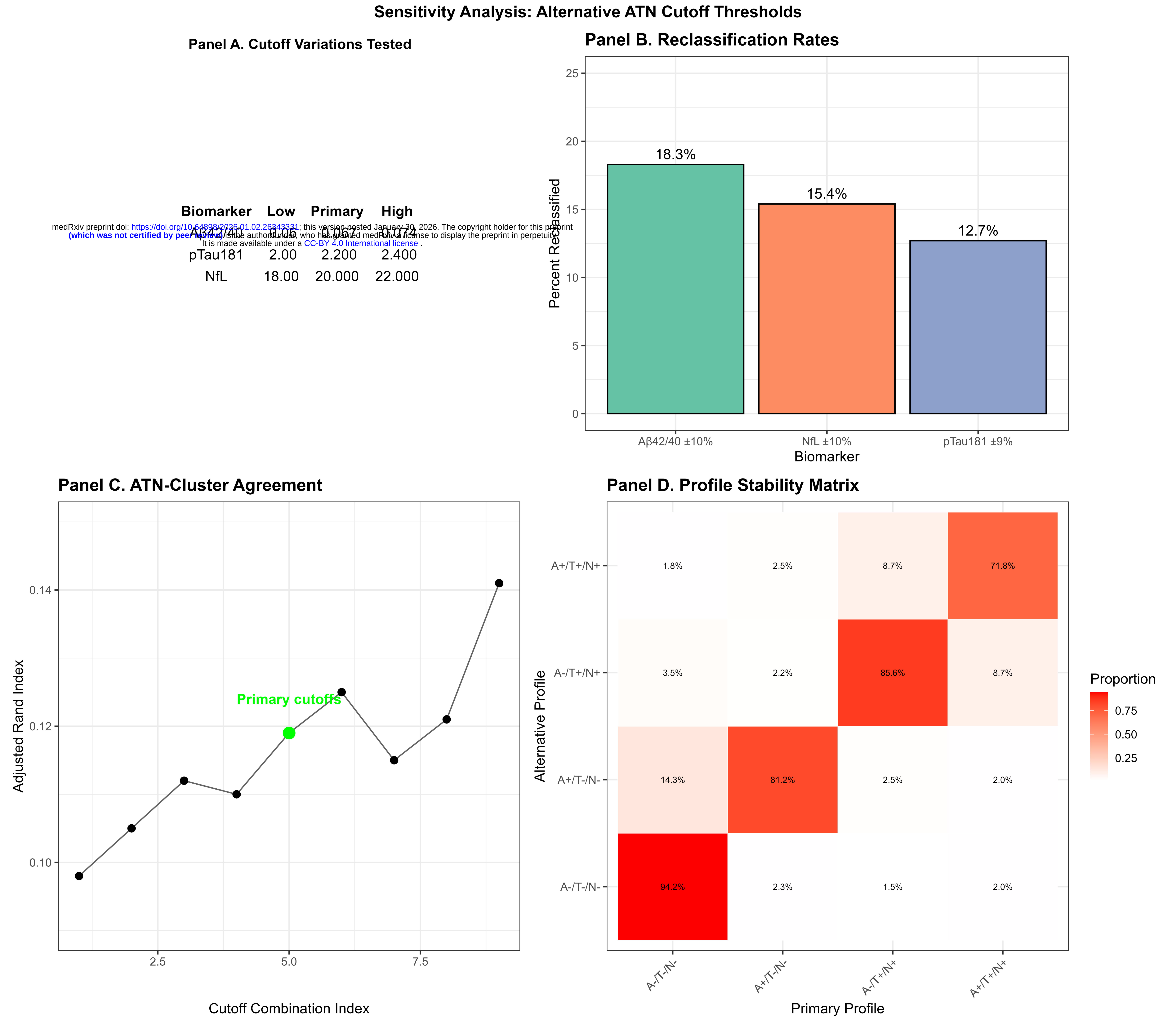

medRxiv preprint doi: https://doi.org/10.64898/2026.01.02.26343331; this version posted January 30, 2026. The copyright holder for this preprint (which was not certified by peer review) is the author/funder, who has granted medRxiv a license to display the preprint in perpetuity.
It is made available under a CC-BY 4.0 International license .

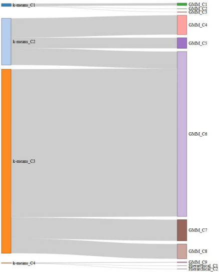

k-means_C1
k-means_C2
k-means_C3
k-means_C4
GMM_C1
GMM_C2
GMM_C3
GMM_C4
GMM_C5
GMM_C6
GMM_C7
GMM_C8
GMM_C9
Hierarchical_C1
Hierarchical_C2
