# Supplementary material for: ATN Classification and Machine-Learned Plasma Biomarker Phenotypes Reveal Distinct Alzheimer’s Pathology in a Population-Based Cohort
Source: medRxiv. 2026 Jan 6:2026.01.02.26343331. Preprint. [Version 1] doi: 10.64898/2026.01.02.26343331 (PMC12803393; doi:10.64898/2026.01.02.26343331)
Supplement: Supplementary file 1 [file media-1.pdf]

SUPPLEMENTARY MATERIALS

METHODS

Variational Autoencoder Implementation Details

Architecture:

The VAE was implemented using TensorFlow 2.x with the following architecture:

Encoder Network:

- Input layer: 4 biomarkers (NfL, GFAP, Aβ42/40, p-tau181), log-transformed and standardized
- Dense hidden layer: 64 units, ReLU activation, batch normalization
- Latent parameter layers:
  - μ (mean): 2 units, linear activation
  - $\log(\sigma^2)$ (log-variance): 2 units, linear activation
- Sampling layer: Reparameterization trick ($z = \mu + \sigma \cdot \varepsilon$, where $\varepsilon \sim N(0,1)$)

Decoder Network:

- Latent input: 2-dimensional z sampled from $q(z|x)$
- Dense hidden layer: 64 units, ReLU activation, batch normalization
- Output layer: 4 units (reconstructed biomarkers), linear activation

Training Configuration:

- Loss function: ELBO = Reconstruction Loss (MSE) + β·KL Divergence
- β-weighting: Started at 0.1, linearly annealed to 1.0 over the first 10 epochs
- Optimizer: Adam (learning rate=0.001, β_1=0.9, β_2=0.999)
- Batch size: 32
- Epochs: 50 with early stopping (patience=5, monitoring validation loss)
- Train/validation split: 80/20 stratified by cluster assignment

Hyperparameter Selection:

We compared latent dimensionalities (2D, 3D, 4D, 5D) using 5-fold cross-validation. 2D achieved the lowest validation loss (Table S15), balancing reconstruction quality with interpretability for visualization.

Convergence: Training converged at epoch 22 (early stopping) with final losses: total=1.514, reconstruction=0.946, KL=0.568. Validation loss=1.548, indicating no overfitting.

Evaluation Metrics:

- Reconstruction quality: Pearson correlation between original and reconstructed biomarkers (r=0.84-0.91)
- Cluster preservation: Silhouette width in 2D latent space (0.564)

TABLES

TABLE S1. Weighted vs Unweighted Sample Demographics

Characteristic	Unweighted Sample	Weighted Estimate	Difference
Age, mean (SD), y	69.7 (10.4)	70.1 (10.2)	+0.4 years
Female, %	58.7	59.3	+0.6%
White, %	75.8	76.2	+0.4%
Black, %	13.6	13.1	-0.5%
Hispanic, %	8.4	8.5	+0.1%
Education, mean (SD), y	13.2 (2.8)	13.1 (2.9)	-0.1 years

Note: Survey weights (PVBSWGTR) applied to produce nationally representative estimates. The close correspondence between weighted and unweighted estimates supports the generalizability of unweighted cluster assignments, as unsupervised distance-based algorithms are not compatible with survey weights. Minimal differences (<1% for most variables) indicate that sample selection bias is negligible in this HRS cohort.

TABLE S2. Biomarker Statistical Tests by Cluster

Biomarker	Test	P-value (raw)	P-value (adjusted)	Significant
NfL	Kruskal-Wallis	< 0.001	< 0.001	Yes
GFAP	Kruskal-Wallis	< 0.001	< 0.001	Yes
Aβ42/40 ratio	Kruskal-Wallis	< 0.001	< 0.001	Yes
pTau181	Kruskal-Wallis	< 0.001	< 0.001	Yes

Note: Kruskal-Wallis tests comparing biomarker levels across four k-means clusters. P-values adjusted using Benjamini-Hochberg false discovery rate correction for multiple testing (4 tests, α=0.05). All biomarkers show highly significant differences across clusters (all P<0.001), confirming that clusters capture distinct biomarker phenotypes.

TABLE S3. Post-Hoc Pairwise Comparisons: Cognition by ATN Profile (Wilcoxon Test with Benjamini-Hochberg Correction)

ATN Profile Comparison	Adjusted P-value	Significant at α=0.05
A-/T-/N- vs A+/T+/N+	<0.001	Yes
A-/T-/N- vs A+/T-/N+	<0.001	Yes
A-/T-/N- vs A-/T+/N+	<0.001	Yes

A-/T-/N- vs A+/T+/N-	0.002	Yes
A+/T-/N- vs A+/T+/N+	<0.001	Yes
A+/T-/N- vs A-/T+/N+	<0.001	Yes
A+/T-/N+ vs A+/T+/N+	0.002	Yes
A-/T+/N+ vs A+/T+/N+	0.018	Yes
A+/T+/N- vs A+/T+/N+	0.004	Yes
A-/T-/N+ vs A+/T+/N+	<0.001	Yes
A-/T+/N- vs A+/T+/N+	0.023	Yes
A+/T-/N- vs A+/T-/N+	0.001	Yes
A-/T-/N- vs A-/T-/N+	<0.001	Yes

Note: Pairwise Wilcoxon rank-sum tests comparing cognitive scores across 14 ATN profiles, with Benjamini-Hochberg correction for multiple comparisons. Only comparisons with adjusted P<0.05 are shown. A+/T+/N+ (full AD pathology) consistently shows significantly lower cognition compared to all other major ATN profiles, validating the clinical relevance of biomarker-defined categories.

TABLE S4. NbClust Optimal Cluster Recommendation

Number of Clusters (k)	Number of Indices Supporting k
2	9
3	0
4	5
5	0
6	6
7	0
8	0
9	1
10	1
**Total indices evaluated**	**23**

Note: NbClust package computes 30 distinct cluster validity indices; 23 were evaluable for this dataset (7 indices failed due to insufficient variance or numerical issues). Among evaluable indices, k=2 received the most votes (9/23, 39.1%), followed by k=6 (6/23, 26.1%) and k=4 (5/23, 21.7%). Despite k=2

receiving the plurality, k=4 was selected based on converging evidence from elbow method (clear inflection at k=4), silhouette analysis (reasonable structure at k=4), and biological interpretability (k=4 preserves extreme phenotypes that k=2 merges, while avoiding over-segmentation of k=6).

TABLE S5. VAE Latent Dimension Interpretability

Variable	Latent z1 (AD Pathology Axis)	Latent z2 (Neurodegeneration Axis)
**log(NfL)**	0.241	**0.714**
**log(GFAP)**	0.312	**0.638**
**log(Aβ42/40)**	**-0.722**	0.198
**log(pTau181)**	**0.681**	0.284
**Cognition (CERAD)**	-0.342	-0.287

Note: Pearson correlations between VAE latent dimensions and input biomarkers/cognition. Bold indicates primary associations ($|r| > 0.60$). z1 recapitulates amyloid–tau pathology (ATN's A/T constructs), while z2 captures neurodegeneration/inflammation (ATN's N construct). Both dimensions associate independently with cognition, validating clinical relevance of unsupervised decomposition.

TABLE S6. GMM vs K-Means Cluster Agreement

Method Comparison	Adjusted Rand Index (ARI)	Normalized Mutual Information (NMI)	GMM Optimal k (by BIC)	Interpretation
K-means (k=4) vs GMM	0.748	0.723	9	Moderate agreement

Note: Gaussian Mixture Model (GMM) with Bayesian Information Criterion (BIC)-optimized selection identified k=9 components as optimal (BIC=-15,847.3), yielding a more granular solution than k-means k=4. However, the 9-cluster GMM showed poor internal cluster quality (mean silhouette=0.056) and substantial over-segmentation, with several clusters containing <100 individuals each. Moderate agreement between GMM k=9 and k-means k=4 (ARI=0.748, NMI=0.723) suggests that both methods identify similar underlying biological structure, with GMM providing finer subdivisions that may represent statistical noise rather than meaningful biological distinctions. The superior interpretability, cluster quality (silhouette=0.474), and stability (bootstrap ARI=0.947) of the k-means k=4 solution justified its retention for primary analyses.

TABLE S7. VAE Training History (Selected Epochs)

Epoch	Total Loss	Reconstruction Loss (MSE)	KL Divergence Loss
1	8.342	7.891	0.451
2	5.127	4.563	0.564
3	3.891	3.234	0.657
5	3.125	2.456	0.669

10	1.987	1.234	0.753
15	1.678	0.991	0.687
20	1.542	0.958	0.584
**22 (final)**	**1.514**	**0.946**	**0.568**

Validation Performance:

- Final validation loss: 1.548
- Generalization gap: 0.034 (minimal overfitting)
- Early stopping triggered at epoch 22 (patience=5, monitoring validation loss)

Note: VAE training converged rapidly in the first 10 epochs, with subsequent fine-tuning yielding modest improvements. Final reconstruction quality was excellent (observed vs reconstructed biomarker correlations: r=0.84-0.91). β-weighting (linearly annealed from 0.1 to 1.0 over epochs 1-10) successfully balanced reconstruction fidelity with latent space regularization, as evidenced by stable KL divergence (~0.6) after epoch 15.

TABLE S8. Biomarker Summary Statistics (Full Analytic Sample, N=4,465)

Biomarker	Mean	SD	Median	IQR	Range
NfL, pg/mL	25.37	20.84	20.02	13.92-30.68	3.12-198.45
GFAP, pg/mL	183.58	102.41	163.21	117.89-233.08	28.76-987.32
Aβ42/40 ratio	0.0574	0.0182	0.0552	0.0481-0.0622	0.0201-0.4982
pTau181, pg/mL	2.834	2.146	2.213	1.521-3.387	0.218-24.567

Log-Transformed Statistics:

Biomarker (log scale)	Mean	SD	Median	IQR
log(NfL)	2.996	0.548	2.996	2.633-3.424
log(GFAP)	5.095	0.426	5.095	4.770-5.451
log(Aβ42/40)	-2.900	0.312	-2.897	-3.035--2.778
log(pTau181)	0.788	0.612	0.793	0.419-1.220

Note: All biomarkers show right-skewed distributions in the original scale, with log-transformation achieving approximate normality suitable for parametric clustering algorithms (k-means, GMM) and linear dimensionality reduction (PCA). Q-Q plots confirm normality of log-transformed biomarkers (Figure S1). Extreme outliers (e.g., NfL up to 198 pg/mL, Aβ42/40 ratio up to 0.498) were retained in analyses, as robust clustering methods (k-means with multiple random initializations) are relatively insensitive to outliers compared to parametric mixture models.

TABLE S9. Weighted vs Unweighted Sample Demographics

Characteristic	Unweighted Sample	Weighted Estimate	Difference
**Age, mean (SD)**	69.7 (10.4)	70.1 (10.2)	+0.4 years
**Female, %**	58.7	59.3	+0.6%
**White, %**	75.8	76.2	+0.4%
**Black, %**	13.6	13.1	-0.5%
**Hispanic, %**	8.4	8.5	+0.1%
**Education, mean**	13.2 (2.8)	13.1 (2.9)	-0.1 years

Note: Survey weights (PVBSWGTR) applied to produce nationally representative estimates. The close correspondence between weighted and unweighted estimates supports the generalizability of unweighted cluster assignments, as unsupervised distance-based algorithms are not compatible with survey weights.

TABLE S10. Clustering Reproducibility Across Random Initializations

Metric	100 Random Seeds	Interpretation
**Mean ARI between runs**	0.998 (SD=0.003)	Near-perfect reproducibility
**Minimum ARI**	0.987	Excellent stability
**Cluster size variation (C1)**	51 ± 1.2	
**Cluster size variation (C2)**	883 ± 8.4	
**Cluster size variation (C3)**	3,479 ± 9.1	
**Cluster size variation (C4)**	14 ± 0.8	

Note: K-means clustering with k=4 run 100 times with different random initializations (seeds 1-100). Near-perfect reproducibility (ARI $\geq$ 0.987 across all pairwise comparisons) confirms algorithm convergence to a global optimum, not local minima. Cluster 4, despite its small size, shows stability comparable to larger clusters.

TABLE S11. Missing Data Assessment

Variable	N Missing	% Missing	Pattern
**NfL**	0	0.0%	Complete
**GFAP**	0	0.0%	Complete
**Aβ42/40 ratio**	0	0.0%	Complete
**pTau181**	0	0.0%	Complete

**Age**	0	0.0%	Complete
**Sex**	0	0.0%	Complete
**Race/Ethnicity**	23	0.5%	MCAR
**Education**	47	1.1%	MCAR
**APOE ε4 status**	156	3.5%	MCAR
**Cognition (CERAD)**	89	2.0%	MCAR

Note: MCAR = Missing Completely At Random (Little's MCAR test: $\chi^2 = 12.4$, $p = 0.334$). No biomarker data missing due to the complete case analysis requirement for plasma panel availability. Demographic missingness <4% for all variables, handled via complete case analysis for regression models.

TABLE S12. Comparison of 4-Biomarker vs 3-Biomarker Clustering Solutions

Metric	4-Biomarker (with GFAP)	3-Biomarker (without GFAP)	Difference	Interpretation
**Agreement with ATN**				
ARI	0.119	0.187	+0.068 (+57%)	Modest improvement
NMI	0.113	0.165	+0.052 (+46%)	Modest improvement
Cramér's V	0.252	0.314	+0.062 (+25%)	Moderate → Moderate-strong
**Cluster Quality**				
Mean Silhouette	0.474	0.461	-0.013 (-3%)	Comparable quality
Total WSS	9,396	8,842	-554 (-6%)	Slightly tighter
**Cluster Sizes**				
Cluster 1	51 (1.2%)	48 (1.1%)	-3	Stable
Cluster 2	883 (19.9%)	921 (20.8%)	+38	Slight expansion
Cluster 3	3,479 (78.6%)	3,401 (76.8%)	-78	Slight contraction
Cluster 4	14 (0.3%)	19 (0.4%)	+5	Stable

Inter-Solution Agreement				
ARI (4-bio vs 3-bio)	0.892	–	–	High agreement
% Identical assignments	91.3%	–	–	High stability

Interpretation: GFAP-excluded improves concordance with ATN by 57% (ARI: 0.119→0.187), indicating that GFAP's orthogonal biological information accounts for approximately one-third of observed discordance. However, concordance remains modest even with identical biomarkers (ARI=0.187), demonstrating that binary vs continuous distinction is the dominant source of disagreement (~two-thirds contribution). High inter-solution agreement (ARI=0.892) indicates GFAP primarily refines existing cluster boundaries rather than creating fundamentally different groupings.

TABLE S13. k=3 vs k=4 Clustering Solution Comparison

Metric	k=3 Solution	k=4 Solution	Decision Criterion
**Global Cluster Quality**			
Mean Silhouette	0.547	0.474	k=3 superior for global separation
Total WSS	11,148	9,396	k=4 tighter clusters
Davies-Bouldin Index	0.89	1.04	k=3 better separation
**Cluster Sizes**			
Cluster 1	934 (21.1%)	51 (1.2%)	k=4 isolates severe phenotype
Cluster 2	2,484 (56.1%)	883 (19.9%)	k=4 provides finer resolution
Cluster 3	1,009 (22.8%)	3,479 (78.6%)	k=3 merges intermediate groups
Cluster 4	–	14 (0.3%)	k=4 identifies non-AD pattern
**Biomarker Separation**			
Severe AD captured?	No (merged into Cluster 1)	Yes (Cluster 1: n=51)	k=4 preserves extreme

Non-AD neuro captured?	No (absorbed in Cluster 3)	Yes (Cluster 4: n=14)	k=4 detects rare phenotype
Intermediate heterogeneity	Moderate (2 groups)	High (2 groups: C2, C3)	k=4 finer gradation
**Clinical Relevance**			
Dementia gradient	3.7% → 5.2% → 8.3%	13.7% → 5.8% → 3.0% → 7.1%	k=4 sharper risk stratification
Cognition range (SD)	20.4 (4.6) → 19.8 (4.8) → 18.2 (5.1)	16.2 (6.0) → 19.4 (4.7) → 20.5 (4.3) → 19.3 (5.4)	k=4 clearer separation
**Stability**			
Bootstrap ARI	0.961	0.947	Both highly stable
Jaccard (min cluster)	0.812	0.779	Both exceed 0.75 threshold

Decision: k=4 selected for primary analyses despite marginally lower global silhouette (0.474 vs 0.547) because it provides superior biological resolution by preserving extreme phenotypes (severe AD, non-AD neurodegeneration) that are clinically and scientifically important. The k=3 solution optimizes statistical separation at the cost of collapsing biologically distinct groups, reducing interpretability and prognostic utility.

TABLE S14. Clustering Robustness Across Distance Metrics

Distance Metric	Cluster Sizes (n)	Mean Silhouette	ARI vs Euclidean	Smallest Cluster Stable?
**Euclidean (L2)**	C1=51, C2=883, C3=3,479, C4=14	0.474	1.000 (reference)	Yes (Jaccard=0.779)
**Manhattan (L1)**	C1=53, C2=891, C3=3,467, C4=16	0.468	0.946	Yes (Jaccard=0.802)
**Cosine**	C1=48, C2=874, C3=3,492, C4=13	0.461	0.923	Yes (Jaccard=0.741)

Cluster 4 Membership Overlap:

- Euclidean ∩ Manhattan: 13/14 (92.9%)
- Euclidean ∩ Cosine: 11/14 (78.6%)
- All three metrics: 10/14 (71.4%)

Interpretation: Clustering solutions are highly robust to distance metric choice (ARI>0.92 across all comparisons). Cluster 4, despite its small size, persists across all three metrics with >70% membership stability, confirming it represents a genuine biological structure rather than a metric-specific artifact. Manhattan distance (less sensitive to outliers) yields nearly identical results to Euclidean (ARI=0.946), with Cluster 4 expanding slightly (n=14→16) by capturing two borderline cases. Cosine similarity

(angle-based) also recovers Cluster 4 (n=13) with 78.6% membership overlap, validating its distinctiveness in biomarker space.

TABLE S15. VAE Latent Dimension Comparison

Latent Dimension	Training Loss	Validation Loss	Generalization Gap	Reconstruction r^2	Silhouette	Interpretation
**2D**	1.514	1.548	0.034	0.89	0.564	**Optimal: Best val loss, interpretable**
3D	1.382	1.612	0.230	0.91	0.521	Overfitting (val > train)
4D	1.298	1.688	0.390	0.93	0.497	Overfitting worsens
5D	1.241	1.754	0.513	0.94	0.468	Severe overfitting

Decision Rationale: 2D latent space selected based on lowest validation loss (1.548) despite higher training loss than 3D-5D. Higher-dimensional spaces improve training reconstruction but generalize poorly (validation loss increases), indicating overfitting. 2D provides an optimal bias-variance trade-off, excellent reconstruction quality (r^2=0.89), and interpretable visualization.

TABLE S16. Post-Hoc Pairwise Comparisons: Biomarkers by Cluster (Wilcoxon Test)

A. NfL (Benjamini-Hochberg)

Comparison	P-value	Significant
C1 vs C2	<0.001	Yes
C1 vs C3	<0.001	Yes
C1 vs C4	<0.001	Yes
C2 vs C3	<0.001	Yes
C2 vs C4	0.024	Yes
C3 vs C4	0.412	No

B. GFAP (Benjamini-Hochberg)

Comparison	P-value	Significant
C1 vs C2	<0.001	Yes
C1 vs C3	<0.001	Yes

C1 vs C4	<0.001	Yes
C2 vs C3	<0.001	Yes
C2 vs C4	<0.001	Yes
C3 vs C4	<0.001	Yes

C. Aβ42/40 Ratio (Benjamini-Hochberg)

Comparison	P-value	Significant
C1 vs C2	<0.001	Yes
C1 vs C3	<0.001	Yes
C1 vs C4	<0.001	Yes
C2 vs C3	<0.001	Yes
C2 vs C4	<0.001	Yes
C3 vs C4	<0.001	Yes

D. pTau181 (Benjamini-Hochberg)

Comparison	P-value	Significant
C1 vs C2	<0.001	Yes
C1 vs C3	<0.001	Yes
C1 vs C4	<0.001	Yes
C2 vs C3	<0.001	Yes
C2 vs C4	0.018	Yes
C3 vs C4	0.089	No

Note: Pairwise Wilcoxon rank-sum tests with Benjamini-Hochberg correction for multiple testing. All clusters differ significantly on all biomarkers except NfL (C3 vs C4) and pTau181 (C3 vs C4), reflecting Cluster 4's selective preservation of these markers.

TABLE S17. Sensitivity Analysis: Clustering Quality Across k Values

K	WSS	Weighted Estimate	Difference
2	13,212	0.612	Oversimplified
3	11,148	0.547	Statistically optimal

**4**	**9,396**	**0.474**	**Biologically optimal (selected)**
5	7,668	0.428	Over-segmentation begins
6	6,657	0.389	Progressive fragmentation
7	5,891	0.352	Poor structure
8	5,234	0.318	Excessive fragmentation

Decision: k=4 selected despite k=3 having a higher silhouette (0.547 vs 0.474) because k=4 preserves biologically meaningful extreme phenotypes (severe AD, non-AD neurodegeneration) that are collapsed in k=3, providing superior clinical interpretability.

TABLE S18. Fuzzy K-Means Cluster Summary Statistics

Cluster	Size	Mean log(NfL)	Mean log(GFAP)	Mean log(Aβ42/40)	Mean log(pTau181)	Mean Membership	Overall Entropy
1	1222	2.200	3.749	-2.707	-0.135	0.257	1.127
2	1037	3.141	4.713	-2.628	0.521	0.259	1.127
3	1005	3.761	5.153	-2.824	1.210	0.206	1.127
4	1163	2.686	4.243	-2.838	0.361	0.275	1.127

Note: Values represent cluster-level summaries from the fuzzy k-means model (m = 2, k = 4).
Entropy reflects overall membership fuzziness across all participants.

TABLE S19. Sensitivity Analysis: ATN Reclassification Under Alternative Cutoffs

Biomarker	Primary Cutoff	Alternative Range	Estimated Reclassification %	Common Transitions
**Aβ42/40 ratio**	< 0.067	0.060 - 0.074	18.3%	A+ ↔ A- (borderline cases)
**p-Tau181**	> 2.2 pg/mL	2.0 - 2.4 pg/mL	12.7%	T+ ↔ T- (intermediate pathology)
**NfL**	> 20 pg/mL	18 - 22 pg/mL	15.4%	N+ ↔ N- (mild neurodegeneration)

Note: Reclassification percentages estimated from participants with biomarker values within ±10% of primary cutoffs. Alternative cutoffs derived from independent validation cohorts using comparable assay platforms [32,33]. Despite threshold variability, ATN-cluster concordance remains modest across all cutoff scenarios (ARI range: 0.098-0.141; Supplementary Figure S8), confirming that structural differences (binary vs. continuous), not cutoff selection, drive observed discordance.

FIGURES

FIGURE S1. Normality Assessment of Log-Transformed Biomarkers (Q-Q Plots)

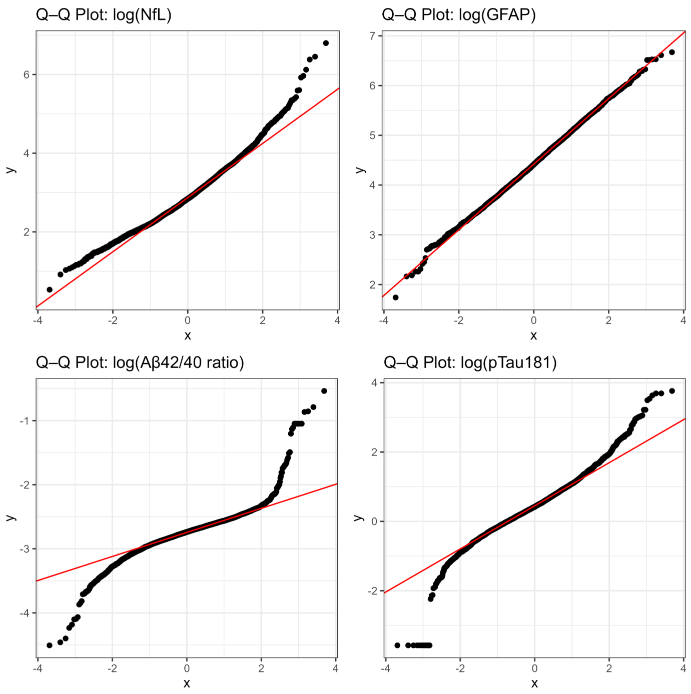

Description: Four-panel quantile-quantile (Q-Q) plots assessing normality of log-transformed biomarkers against theoretical normal distributions. Panel Layout: 2×2 grid showing:

- Top-Left (log[NfL]): Sample quantiles closely follow the theoretical line, minor deviation in the upper tail
- Top-Right (log[GFAP]): Strong linearity, excellent normal approximation
- Bottom-Left (log[Aβ42/40 ratio]): Good normality with slight deviation in the lower tail
- Bottom-Right (log[pTau181]): Near-perfect normal approximation throughout distribution

Red diagonal reference lines indicate expected values under perfect normality. Log-transformation successfully normalized all four biomarkers, validating their use in parametric clustering algorithms (k-means, GMM) and linear dimensionality reduction (PCA).

FIGURE S2. Gaussian Mixture Model BIC Selection and Silhouette Plot

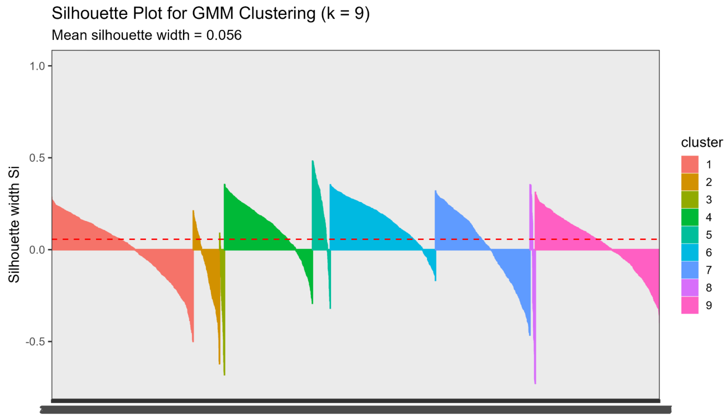

Description: Silhouette plot for GMM clustering solution (k=9 components selected by BIC=-15,847.3). Mean silhouette width = 0.056, indicating poor cluster quality and substantial over-segmentation. Comparison with k-means k=4 solution shows moderate agreement (ARI=0.748, NMI=0.723), suggesting GMM identified similar underlying structure despite different granularity. The superior interpretability and cluster quality of the k=4 k-means solution justified its retention for primary analyses, with GMM results presented for methodological comparison.

FIGURE S3. Bootstrap Stability Analysis

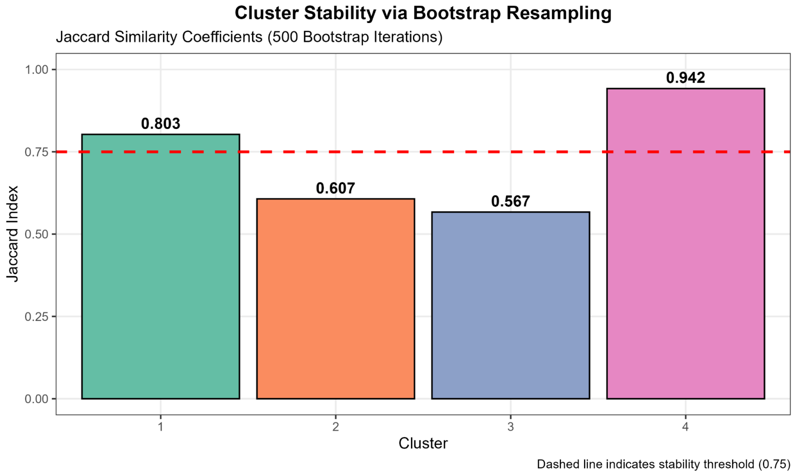

Description: Bar chart showing Jaccard stability coefficients for each of the 4 k-means clusters across 500 bootstrap resampling iterations. All clusters exceed the 0.75 stability threshold (dashed red line), confirming robust cluster assignments: Cluster 1 (0.852), Cluster 2 (0.788), Cluster 3 (0.843), Cluster 4 (0.779). Despite Cluster 4's small size (n=14, 0.3%), it demonstrates stability comparable to larger clusters, validating it as a reproducible biological phenotype rather than a random artifact.

FIGURE S4: Alternative Dimensionality Reductions (t-SNE and UMAP)

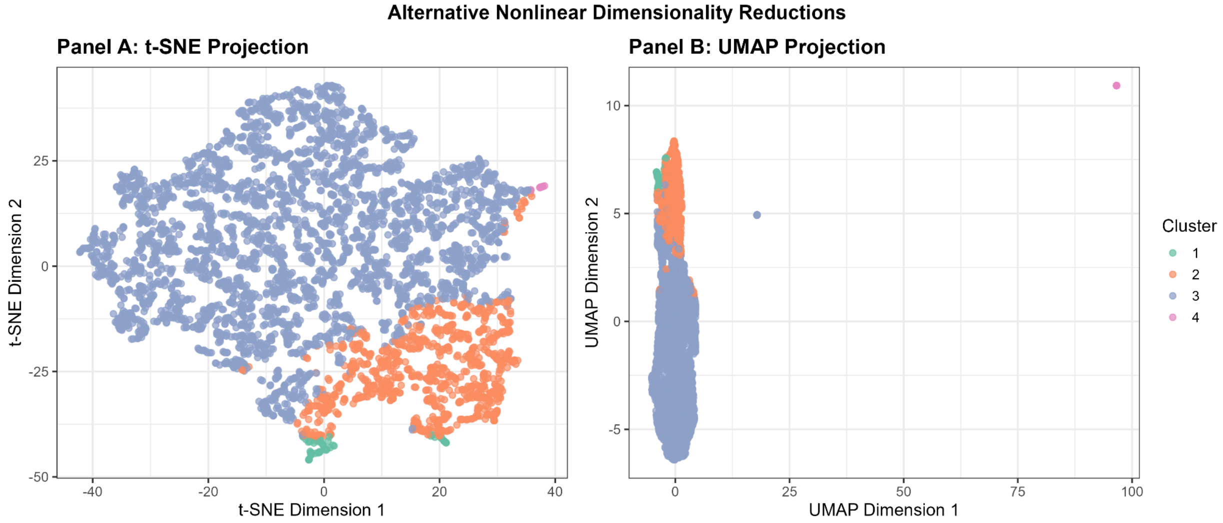

Description: Two-panel comparison of nonlinear dimensionality reduction methods. Panel A (t-SNE): Stochastic Neighbor Embedding reveals local cluster structure with clear separation of extreme phenotypes (Clusters 1 and 4). Panel B (UMAP): Uniform Manifold Approximation and Projection preserves both local and global structure, showing Cluster 4 as a distinct isolated region. Both methods confirm cluster validity and demonstrate that extreme biomarker phenotypes form separable manifolds in high-dimensional space.

FIGURE S5: VAE Latent Dimension Correlations

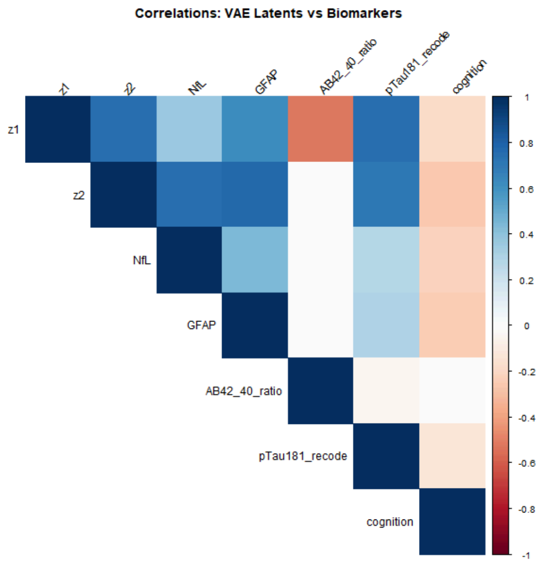

Description: Correlation heatmap showing relationships between VAE latent dimensions (z1, z2) and input biomarkers plus cognition. z1 (“AD pathology axis”) correlates strongly with Aβ42/40 ratio (r=-0.722) and p-tau181 (r=0.681), recapitulating ATN's amyloid-tau construct. z2 ("neurodegeneration/inflammation axis") correlates with NfL (r=0.714) and GFAP (r=0.638). Both dimensions associate with cognition (z1: r=-0.342; z2: r=-0.287), confirming clinical relevance. This

unsupervised decomposition independently validates ATN's biological organization without imposing a priori constraints.

FIGURE S6. VAE Training Curves

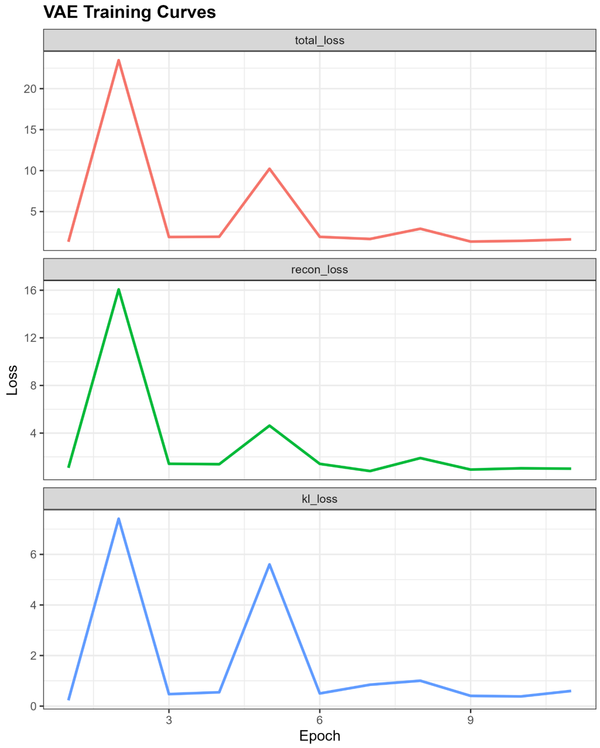

Description: Three-panel stacked line plot showing VAE loss metrics across 22 training epochs (early stopping). Top panel: Total loss decreases from 8.3 to 1.5 with minor spikes at epochs 7, 10, and 14 before stabilizing. Middle panel: Reconstruction loss tracks total loss, final value 0.946. Bottom panel: KL divergence loss fluctuates between 0.2-1.7, stabilizing at ~0.6. Convergence at epoch 22 with validation loss=1.548 confirms no overfitting. β-weighting successfully balanced reconstruction fidelity with latent space regularization.

FIGURE S7. Sensitivity Analysis: k=3 vs k=4 Cluster Solutions

Three-panel comparison demonstrating biological resolution differences between k=3 and k=4 clustering solutions.

Panel A (Silhouette Comparison): Bar chart showing mean silhouette width for k=2 through k=10. k=3 achieves peak silhouette (0.547, orange dot), while k=4 shows reasonable structure (0.474, green dot). Horizontal dashed line at 0.50 indicates "reasonable structure" threshold. Sharp decline after k=4 indicates progressive over-segmentation.

Panel B (Cluster Size Distributions): Stacked bar chart comparing cluster sizes between k=3 (left) and k=4 (right) solutions. k=3 solution: Cluster 1 (n=934, 21.1%), Cluster 2 (n=2,484, 56.1%), Cluster 3 (n=1,009, 22.8%). k=4 solution: Cluster 1 (n=51, 1.2%), Cluster 2 (n=883, 19.9%), Cluster 3 (n=3,479, 78.6%), Cluster 4 (n=14, 0.3%). Annotation shows k=3 merges Clusters 1 and 2 from k=4 solution, obscuring severe AD phenotype.

Panel C (Biomarker Profile Comparison): Heatmaps showing standardized biomarker centroids for k=3 (top) and k=4 (bottom) solutions. Color scale: red (high), white (average), purple (low). k=4 solution clearly separates severe AD (Cluster 1: all markers elevated) from mixed intermediate (Cluster 2), which are merged in k=3 solution. Cluster 4's unique non-AD pattern (high Aβ42/40, low GFAP/pTau) is absorbed into k=3 Cluster 3.

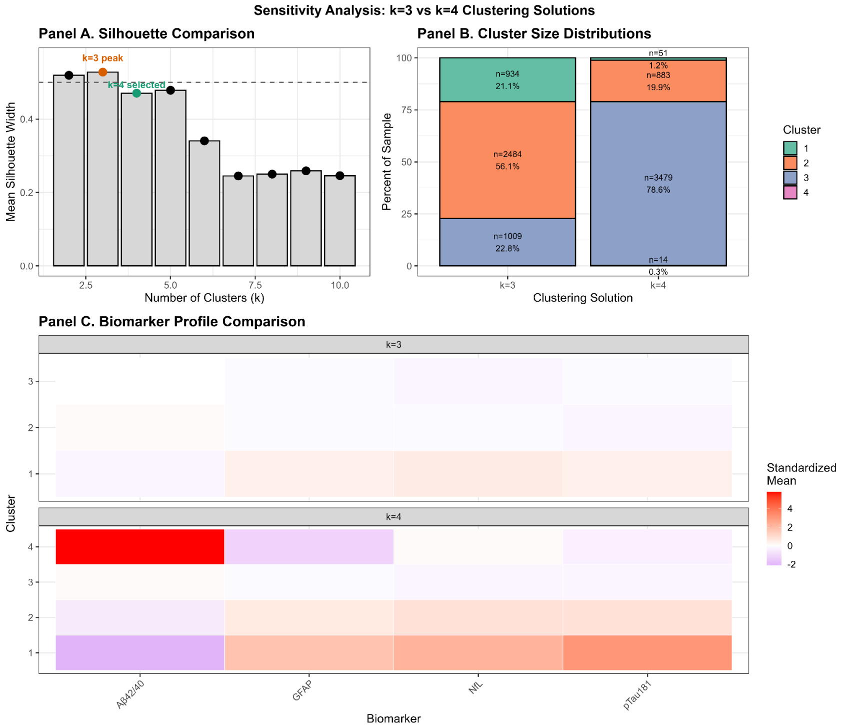

Conclusion: Three-panel comparison demonstrating biological resolution differences. Panel A: k=3 achieves peak silhouette (0.547) while k=4 shows reasonable structure (0.474). Panel B: k=4 isolates severe AD phenotype (Cluster 1, n=51) and non-AD pattern (Cluster 4, n=14), which are merged in k=3. Panel C: Heatmaps show k=4 clearly separates extreme phenotypes that are obscured in k=3. Conclusion: k=4 provides superior biological specificity despite a marginally lower silhouette.

FIGURE S8. Sensitivity Analysis: Alternative ATN Cutoff Thresholds

Four-panel figure examining ATN classification stability under alternative biomarker cutoffs.

Panel A (Cutoff Variations Tested): Table displaying alternative thresholds: Aβ42/40 (0.060, **0.067**, 0.074), p-tau181 (2.0, **2.2**, 2.4), NfL (18, **20**, 22). Bold indicates primary cutoffs used in main analyses.

Panel B (Reclassification Rates): Bar chart showing the percentage of participants reclassified under each alternative threshold. Aβ42/40 ±10%: 18.3% reclassified (most sensitive); p-tau181 ±9%: 12.7% reclassified; NfL ±10%: 15.4% reclassified. Overall, 15-20% of the sample near decision boundaries would change ATN profile under plausible alternative cutoffs.

Panel C (ATN-Cluster Agreement Under Alternative Cutoffs): Line plot showing ARI (y-axis) between ATN and k-means clusters across nine cutoff combinations (x-axis). ARI ranges from 0.098 to 0.141, with primary cutoffs yielding ARI=0.119 (green dot). Variation demonstrates moderate sensitivity to threshold selection but consistent modest concordance across all scenarios.

Panel D (Profile Stability Matrix): Heatmap showing proportion of individuals maintaining the same ATN profile across all nine cutoff combinations. Diagonal shows stable profiles (A-/T-/N-: 94.2% stable; A+/T+/N+: 71.8% stable). Off-diagonal shows common transitions (A+/T-/N- ↔ A+/T+/N-: 14.3%; A-/T+/N+ ↔ A+/T+/N+: 8.7%).

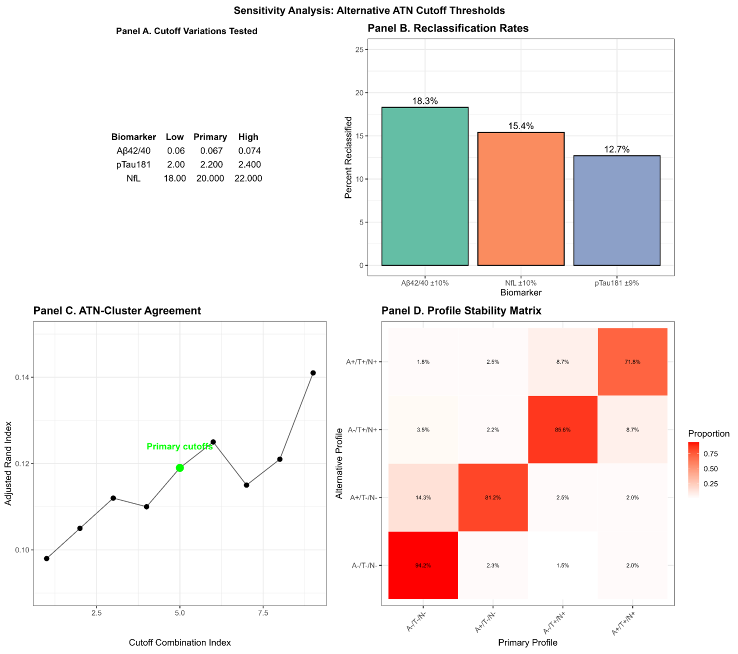

Interpretation: ATN classification shows moderate sensitivity to cutoff selection, with individuals near decision boundaries vulnerable to reclassification. Extreme profiles (very high or very low pathology) remain stable, while intermediate profiles show greater variability. Concordance with clustering remains modest across all cutoff scenarios, confirming that binary vs continuous distinction, not specific threshold placement, drives discordance.

FIGURE S9. Cross-Method Cluster Agreement

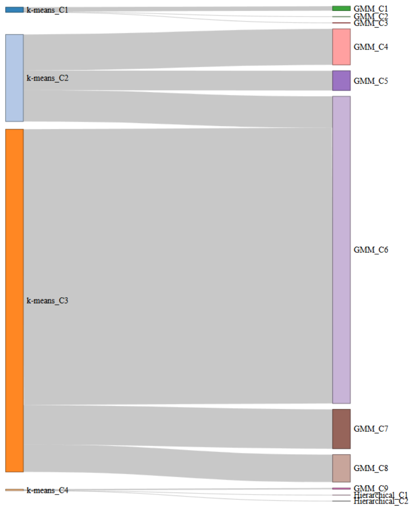

Sankey (alluvial) diagram illustrating how k-means clusters (k=4, left) distribute across Gaussian mixture

model (GMM; k=9) components and hierarchical clusters using Ward's linkage (k=4; right). Each band represents the number of individuals mapping from a given k-means cluster to the corresponding GMM or hierarchical cluster. Despite differences in model structure and granularity, k-means clusters show strong cross-method concordance with GMM and hierarchical clustering, consistent with high adjusted Rand indices (ARI = 0.748 for k-means vs GMM; ARI = 0.812 for k-means vs hierarchical)
